# Supplementary material for: Wnt signaling modulates the response to DNA damage in the Drosophila wing imaginal disc by regulating the EGFR pathway
Source: PLoS Biol. 2024 Jul 24;22(7):e3002547. doi: 10.1371/journal.pbio.3002547 (PMC11341097; doi:10.1371/journal.pbio.3002547)
Supplement: S1 Table — (DOCX) [file pbio.3002547.s001.docx]

**Table S1. Sources and genotypes of *Drosophila* lines used in this study**

| **Fly Line** | **Source** | **Full Genotype** |
| --- | --- | --- |
| *hh-Gal4, tubGal80ts > UAS:Cas9.P2* | Perrimon TRiP-KO toolbox | *w[*]; P{y[+t7.7] w[+mC]=UAS-Cas9.P2}attP40, P{w[+mC]=tubP-GAL80[ts]}10]/CyO; P{w[+mC]=hh-Gal4}/TM6B, Tb[1]* |
| *hh-Gal4 > UAS:Cas9.P2* | Perrimon TRiP-KO toolbox | *w[*]; P{y[+t7.7] w[+mC]=UAS-Cas9.P2}attP40/CyO; P{w[+mC]=hh-Gal4}/TM6B, Tb[1]* |
| *nub-Gal4 > UAS:Cas9.2* | BL67086 | *w[*]; P{w[+mW.hs]=GawB}nubbin-AC-62; P{y[+t7.7] w[+mC]=UAS-Cas9.P2}attP2* |
| *hh-Gal4 > UAS:u[M]Cas9* | UAS:u[M]Cas9 of Fillip Port | *w[*]; P{y[+t7.7] w[+mC]=UAS-uMCas9}attP40 / CyO ; P{w[+mC]=hh-Gal4}/TM6B, Tb[1]* |
| *en-Gal4, tubGal80[ts], UAS:GFP > UAS:dCas9-VPR* | BL67069 | *w[*]; P{w[+mW.hs]=en2.4-GAL4}e16E, P{w[+mC]=UAS-2xEGFP}AH2/CyO; P{y[+t7.7] w[+mC]=UAS-3xFLAG.dCas9.VPR}attP2, P{w[+mC]=tubP-GAL80[ts]}2* |
| *mCherry RNAi* | BL35785 | *y[1] sc[*] v[1] sev[21]; P{y[+t7.7] v[+t1.8]=VALIUM20-mCherry.RNAi}attP2* |
| *wg RNAi* | BL32994 | *y[1] sc[*] v[1] sev[21]; P{y[+t7.7] v[+t1.8]=TRiP.HMS00794}attP2* |
| *arm RNAi* | BL35004 | *y[1] sc[*] v[1] sev[21]; P{y[+t7.7] v[+t1.8]=TRiP.HMS01414}attP2* |
| *UAS-wg* | BL5918 | *w[*];; P{w[+mC]=UAS-wg.H.T:HA1}6C* |
| *Chk1/grps RNAi-1* | BL36685 | *y[1] sc[*] v[1] sev[21]; P{y[+t7.7] v[+t1.8]=TRiP.HMS01573}attP2* |
| *Chk1/grps RNAi-2* | BL27277 | *y[1] v[1]; P{y[+t7.7] v[+t1.8]=TRiP.JF02588}attP2* |
| *ATR/mei-41 RNAi* | BL35371 | *y[1] sc[*] v[1] sev[21]; P{y[+t7.7] v[+t1.8]=TRiP.GL00284}attP2* |
| *ATM/tefu RNAi* | BL44417 | *y[1] sc[*] v[1] sev[21]; P{y[+t7.7] v[+t1.8]=TRiP.GL00138}attP2* |
| *Chk2/lok RNAi* | BL35152 | *y[1] sc[*] v[1] sev[21]; P{y[+t7.7] v[+t1.8]=TRiP.GL00020}attP2* |
| *p53[DN]* | BL8421 | *y[1] w[1118]; P{w[+mC]=UAS-p53.H159N.Ex}3* |
| *p53 RNAi* | BL29351 | *y[1] v[1]; P{y[+t7.7] v[+t1.8]=TRiP.JF02513}attP2* |
| *E2F1 RNAi* | BL36126 | *y[1] sc[*] v[1] sev[21]; P{y[+t7.7] v[+t1.8]=TRiP.HMS01541}attP2* |
| *UAS:Rbf* | BL50747 | *w[*]; P{w[+mC]=UAS-Rbf.D}III* |
| *cycA RNAi* | BL35694 | *y[1] sc[*] v[1] sev[21]; P{y[+t7.7] v[+t1.8]=TRiP.GLV21059}attP2* |
| *UAS:yki[3SA]* | BL28817 | *w[*]; P{y[+t7.7] w[+mC]=UAS-yki.S111A.S168A.S250A.V5}attP2* |
| *UAS:EGFR[gammaTop]* | BL59843 | *w[*]; P{w[+mC]=UAS-Egfr.lambdatop}3/TM6C, Sb[1]* |
| *UAS:Hh[GFP]* | BL81025 | *w[*]; P{w[+mC]=UAS-hh.EGFP.H}3/TM3, Sb[1]* |
| *ci RNAi* | BL64928 | *y[1] sc[*] v[1] sev[21]; P{y[+t7.7] v[+t1.8]=TRiP.HMC05801}attP2* |
| *UAS:dpp* | BL1486 | *y[1] w[*]; P{w[+mC]=UAS-dpp.S}42B.4* |
| *UAS:tkv[ACT]* | BL36536 | *y[1] w[*]; P{w[+mC]=UAS-tkv.Q253D.Nb}3/TM3, Sb[1] Ser[1]* |
| *UAS:Bsk[DN]* | BL9311 | *w[*]; P{w[+mC]=UAS-bsk.K53R}20.1a* |
| *UAS:upd* | Perrimon Lab Stock | *w[*]; P9(UAS-upd)26.2/CyO* |
| *Stat92E RNAi* | BL33637 | *y[1] v[1]; P{y[+t7.7] v[+t1.8]=TRiP.HMS00035}attP2* |
| *UAS:Notch[ACT]* | BL26674 | *y[1] w[*]; P{w[+mC]=UAS-Delta::N.DeltaECN}B2a3* |
| *Notch RNAi* | BL33611 | *y[1] v[1]; P{y[+t7.7] v[+t1.8]=TRiP.HMS00001}attP2* |
| *UAS:Myc[ACT]* | BL64759 | *y[1] w[*]; M{w[+mC]=UAS-Myc.HA.WT}ZH-86Fb* |
| *Myc RNAi* | BL36123 | *y[1] sc[*] v[1] sev[21]; P{y[+t7.7] v[+t1.8]=TRiP.HMS01538}attP2* |
| *rolled/ERK RNAi* | BL34855 | *y[1] sc[*] v[1] sev[21]; P{y[+t7.7] v[+t1.8]=TRiP.HMS00173}attP2* |
| *UAS-vn* | Gift of A. Simcox | *UAS-vn (X)* |
| *UAS-Krn* | Gift of A. Simcox | *w;; UAS:s-Krn (III)* |
| *UAS-grk* | Gift of A. Simcox | *w ;; UAS:s-grk (III)* |
| *rho RNAi* | BL56950 | *y[1] v[1]; P{y[+t7.7] v[+t1.8]=TRiP.JF03106}attP2* |
| *UAS:rho* | Gift of M. Freeman | *w;; UAS-rhomboid* |
| *ex-LacZ* | BL44248 | *w[*]; P{w[+mC]=lacW}ex[697]/CyO; TM2/TM6B, Tb[1]* |
| *puc-LacZ* | Gift of K. Kim, Perrimon Lab | *w ; Sp/CyO ; puc-LacZ* |
| *UAS:puc* | BL98328 | *y[1] w[*]; P{w[+mC]=UAS-puc.M}3* |
| *hid RNAi* | VDRC - GD8269 | *w1118;; P{GD8269}* |
| *hid RNAi* | VDRC - GD7912 | *w1118;; P{GD7912}* |
| *hid-EGFP* | BL50751 | *w[*]; P{w[+mC]=hid-EGFP.5'F-WT}3/TM3, P{w[+mC]=ActGFP}JMR2, Ser[1]* |
| *grim RNAi* | VDRC - GD21830 | *w1118; P{GD21830}* |
| *rpr RNAi* | VDRC - GD12050 | *w1118;;P{GD12050}* |
| *rpr RNAi* | VDRC - KK101234 | *w1118 ; P{KK101234}30B* |
| *rpr RNAi* | BL51846 | *y[1] v[1]; P{y[+t7.7] v[+t1.8]=TRiP.HMC03419}attP40* |
| *skl RNAi* | VDRC - GD7172 | *w1118;;P{GD7172}* |
| *skl RNAi* | VDRC - KK102512 | *w1118;;P{KK102512}* |
| *skl RNAi* | BL28678 | *y[1] v[1]; P{y[+t7.7] v[+t1.8]=TRiP.JF03093}attP2* |
| *UAS:p53* | Gift of Leonard Rabinow | *w ; UAS:p53 / CyO ; MKRS / TM6b* |
