# Supplementary material for: Wnt signaling modulates the response to DNA damage in the Drosophila wing imaginal disc by regulating the EGFR pathway
Source: PLoS Biol. 2024 Jul 24;22(7):e3002547. doi: 10.1371/journal.pbio.3002547 (PMC11341097; doi:10.1371/journal.pbio.3002547)
Supplement: S2 Table — (DOCX) [file pbio.3002547.s002.docx]

**Table S2. *Drosophila* sgRNA lines used in this study**

| **sgRNA lines** | **Integration site** | **Genotype** | sgRNA sequence(s) | **Reference** |
| --- | --- | --- | --- | --- |
| *pCFD6-intergenic[2x]* | attP40 | *w{*] ; P{y[+t7.7] w[+mC]=UAS-intergenic[2x].pCFD6}attP40/CyO* | GCAGACAACTGTGATGGCTC, CAGGACTTTATCGCCCAGCA | Ewen-Campen *et al.* 2020 |
| *pCFD6-intergenic[2x]* | attP2 | *w{*] ;; P{y[+t7.7] w[+mC]=UAS-intergenic[2x].pCFD6}attP2/TM6b* | GCAGACAACTGTGATGGCTC, CAGGACTTTATCGCCCAGCA | Ewen-Campen *et al.* 2020 |
| *pCFD6-non-targeting[2x]* | attP2 | *w{*] ;; P{y[+t7.7] w[+mC]=UAS-QUAS[2x].pCFD6}attP2/TM6b* | CTCGGGTAATCGCTTATCCT, CGGATAAACAATTATCCTCA | This study |
| *pCFD6-wg[2x]+intergenic-1[2x]* | attP40 | *w{*] ; P{y[+t7.7] w[+mC]=UAS-wg[2x]-intergenic[2x].pCFD6}attP40/CyO* | GGGGCCGGGGCTCCATGTGGTGG, CGATCCACTCTACGTTGAGAAGG, GCAGACAACTGTGATGGCTC, CAGGACTTTATCGCCCAGCA | This study |
| *pCFD6-wg[2x]+intergenic-1[2x]* | attP2 | *w{*] ;; P{y[+t7.7] w[+mC]=UAS-wg[2x]-intergenic[2x].pCFD6}attP2/TM6b* | GGGGCCGGGGCTCCATGTGGTGG, CGATCCACTCTACGTTGAGAAGG, GCAGACAACTGTGATGGCTC, CAGGACTTTATCGCCCAGCA | This study |
| *pCFD6-wg[2x]+intergenic-2[2x]* | attP40 | *w{*] ; P{y[+t7.7] w[+mC]=UAS-wg[2x]-intergenic-2[2x].pCFD6}attP40/CyO* | GGGGCCGGGGCTCCATGTGGTGG, CGATCCACTCTACGTTGAGAAGG, AGCGGCTATCGTTTAGTTCC, TCAGGTGTGACCCATGCATT | This study |
| *pCFD6-wnt2[2x]-intergenic-1[2x]* | attP40 | *w{*] ; P{y[+t7.7] w[+mC]=UAS-wnt2[2x]-intergenic-1[2x].pCFD6}attP40/CyO* | CGCTGGCCCGGGGTCAGGCC, AATCTACATACTCTGGATTA, GCAGACAACTGTGATGGCTC, CAGGACTTTATCGCCCAGCA | This study |
| *pCFD6-wnt10[2x]-intergenic-1[2x]* | attP40 | *w{*] ; P{y[+t7.7] w[+mC]=UAS-wnt10[2x]-intergenic-1[2x].pCFD6}attP40/CyO* | ATGGCGCGATGTGGCCGGTG, CAGAAGCAGCAGCAAGAAGC, GCAGACAACTGTGATGGCTC, CAGGACTTTATCGCCCAGCA | This study |
| *pCFD6-evi-pFP854* | attP40 | *w{*] ; P{y[+t7.7] w[+mC]=UAS-evi-pFP854.pCFD6}attP40/CyO* | GGCCACTTTGCTGCTCTGCC, GCTGAACTTGCGTTTCCCGC | Gift of Fillip Port & Simon Bullock |
| *Wnt double sgRNAs (collection)* | attP40 | See Ewen-Campen *et al.* 2020 | See Ewen-Campen et al. 2020 | Ewen-Campen *et al.* 2020 |
| *pCFD6-yellow-pFB407* | attP40 | *w{*] ; P{y[+t7.7] w[+mC]=UAS-yellow-pFB407.pCFD6}attP40/CyO* | GCGATATAGTTGGAGCCAGC | This study; sgRNA design from Fillip Port |
| *pCFD6-ebony-pFP545* | attP40 | *w{*] ; P{y[+t7.7] w[+mC]=UAS-ebony-pFB545.pCFD6}attP40/CyO* | gTGGCCATCTGGAAGGCTGG | This study; sgRNA design from Fillip Port |
| *pCFD6-ebony-pFP578* | attP40 | *w{*] ; P{y[+t7.7] w[+mC]=UAS-ebony-pFB578.pCFD6}attP40/CyO* | gATCGAGTCCACGAAGGTTA | This study; sgRNA design from Fillip Port |
| *pCFD6-oskar* | attP40 | *w{*] ; P{y[+t7.7] w[+mC]=UAS-oskar.pCFD6}attP40/CyO* | CTCAGCGGTCACATTGGGAA | This study |
| *pCFD4-wg-CRISPRa* | attP40 | *y[1] sc[*] v[1] sev[21]; P{y[+t7.7] v[+t1.8]=TOE.GS00125}attP40* | ATGAGGTTGCGCAAATAATC, GGAAATGGAAAAACTCTGCC | BL67545 |
