# Supplementary material for: Wnt signaling modulates the response to DNA damage in the Drosophila wing imaginal disc by regulating the EGFR pathway
Source: PLoS Biol. 2024 Jul 24;22(7):e3002547. doi: 10.1371/journal.pbio.3002547 (PMC11341097; doi:10.1371/journal.pbio.3002547)
Supplement: S3 Table — (DOCX) [file pbio.3002547.s003.docx]

**Table S3. Genotype table**

| **Figure** | **Panel** | **Short-hand** | **Full genotype** |
| --- | --- | --- | --- |
| Figure 1 | A; left | hh-Gal4, tubGal80[ts] > Cas9.P2, mCherry RNAi + non-targeting sgRNA | *w ; P{y[+t7.7] w[+mC]=UAS-QUAS[2x].pCFD6}attP40 / P{y[+t7.7] w[+mC]=UAS-Cas9.P2}attP40, P{w[+mC]=tubP-GAL80[ts]}10] ; P{y[+t7.7] v[+t1.8]=VALIUM20-mCherry.RNAi}attP2 / P{w[+mC]=hh-Gal4}* |
|  | A; second from left | hh-Gal4, tubGal80[ts] > Cas9.P2, mCherry RNAi + intergenic sgRNA | *w ; P{y[+t7.7] w[+mC]=UAS-intergenic[2x].pCFD6}attP40 / P{y[+t7.7] w[+mC]=UAS-Cas9.P2}attP40, P{w[+mC]=tubP-GAL80[ts]}10] ; P{y[+t7.7] v[+t1.8]=VALIUM20-mCherry.RNAi}attP2 / P{w[+mC]=hh-Gal4}* |
|  | A; third from left | hh-Gal4, tubGal80[ts] > Cas9.P2, wg RNAi + non-targeting sgRNA | *w ; P{y[+t7.7] w[+mC]=UAS-QUAS[2x].pCFD6}attP40 / P{y[+t7.7] w[+mC]=UAS-Cas9.P2}attP40, P{w[+mC]=tubP-GAL80[ts]}10] ; P{y[+t7.7] v[+t1.8]=TRiP.HMS00794}attP2 / P{w[+mC]=hh-Gal4}* |
|  | A; fourth from left | hh-Gal4, tubGal80[ts] > Cas9.P2, wg RNAi + intergenic sgRNA | *w ; P{y[+t7.7] w[+mC]=UAS-intergenic[2x].pCFD6}attP40 / P{y[+t7.7] w[+mC]=UAS-Cas9.P2}attP40, P{w[+mC]=tubP-GAL80[ts]}10] ; P{y[+t7.7] v[+t1.8]=TRiP.HMS00794}attP2 / P{w[+mC]=hh-Gal4}* |
|  | A; fifth from left | hh-Gal4, tubGal80[ts] > Cas9.P2, arm RNAi + non-targeting sgRNA | *w ; P{y[+t7.7] w[+mC]=UAS-QUAS[2x].pCFD6}attP40 / P{y[+t7.7] w[+mC]=UAS-Cas9.P2}attP40, P{w[+mC]=tubP-GAL80[ts]}10] ; P{y[+t7.7] v[+t1.8]=TRiP.HMS01414}attP2 / P{w[+mC]=hh-Gal4}* |
|  | A; right | hh-Gal4, tubGal80[ts] > Cas9.P2, arm RNAi + intergenic sgRNA | *w ; P{y[+t7.7] w[+mC]=UAS-intergenic[2x].pCFD6}attP40 / P{y[+t7.7] w[+mC]=UAS-Cas9.P2}attP40, P{w[+mC]=tubP-GAL80[ts]}10] ; P{y[+t7.7] v[+t1.8]=TRiP.HMS01414}attP2 / P{w[+mC]=hh-Gal4}* |
|  | A; bottom left | hh-Gal4, tubGal80[ts] > Cas9.P2, UAS-wg + non-targeting sgRNA | *w ; P{y[+t7.7] w[+mC]=UAS-QUAS[2x].pCFD6}attP40 / P{y[+t7.7] w[+mC]=UAS-Cas9.P2}attP40, P{w[+mC]=tubP-GAL80[ts]}10] ; P{w[+mC]=UAS-wg.H.T:HA1}6C / P{w[+mC]=hh-Gal4}* |
|  | A; bottom right | hh-Gal4, tubGal80[ts] > Cas9.P2, UAS-wg + intergenic sgRNA | *w ; P{y[+t7.7] w[+mC]=UAS-intergenic[2x].pCFD6}attP40 / P{y[+t7.7] w[+mC]=UAS-Cas9.P2}attP40, P{w[+mC]=tubP-GAL80[ts]}10] ; P{w[+mC]=UAS-wg.H.T:HA1}6C / P{w[+mC]=hh-Gal4}* |
|  |  |  |  |
| Figure 2 | A; mCherry RNAi | hh-Gal4 > mCherry RNAi | *w / y[1] sc[*] v[1] sev[21] ; P{y[+t7.7] v[+t1.8]=VALIUM20-mCherry.RNAi}attP2 / P{w[+mC]=hh-Gal4}/* |
|  | A; wg RNAi | hh-Gal4 > wg RNAi | *w / y[1] sc[*] v[1] sev[21] ; P{y[+t7.7] v[+t1.8]=TRiP.HMS00794}attP2 / P{w[+mC]=hh-Gal4}* |
|  | B; mCherry RNAi | hh-Gal4, tubGal80[ts] > mCherry RNAi | *w / y[1] sc[*] v[1] sev[21] ; P{w[+mC]=tubP-GAL80[ts]}10]; P{y[+t7.7] v[+t1.8]=VALIUM20-mCherry.RNAi}attP2 / P{w[+mC]=hh-Gal4}* |
|  | B; arm RNAi | hh-Gal4, tubGal80[ts] > arm RNAi | *w / y[1] sc[*] v[1] sev[21] ; P{w[+mC]=tubP-GAL80[ts]}10]; P{y[+t7.7] v[+t1.8]=TRiP.HMS01414}attP2 / P{w[+mC]=hh-Gal4}* |
|  |  |  |  |
| Figure 3 | B; left | hh-Gal4, tubGal80[ts] > Cas9.P2, non-targeting sgRNA | *w ; P{y[+t7.7] w[+mC]=UAS-QUAS[2x].pCFD6}attP40 / P{y[+t7.7] w[+mC]=UAS-Cas9.P2}attP40, P{w[+mC]=tubP-GAL80[ts]}10] ; P{w[+mC]=hh-Gal4}* |
|  | B; right | hh-Gal4, tubGal80[ts] > Cas9.P2, wg+intergenic sgRNA | *w ; P{y[+t7.7] w[+mC]=UAS-wg[2x]-intergenic[2x].pCFD6}attP40 / P{y[+t7.7] w[+mC]=UAS-Cas9.P2}attP40, P{w[+mC]=tubP-GAL80[ts]}10] ; P{w[+mC]=hh-Gal4}* |
|  | C; Chk2 | hh-Gal4, tubGal80[ts] > Cas9.P2, wg+intergenic sgRNA + Chk2 RNAi | *w ; P{y[+t7.7] w[+mC]=UAS-wg[2x]-intergenic[2x].pCFD6}attP40 / P{y[+t7.7] w[+mC]=UAS-Cas9.P2}attP40, P{w[+mC]=tubP-GAL80[ts]}10] ; P{w[+mC]=hh-Gal4} / P{y[+t7.7] v[+t1.8]=TRiP.GL00020}attP2* |
|  | C; p53DN | hh-Gal4, tubGal80[ts] > Cas9.P2, wg+intergenic sgRNA + p53DN | *w ; P{y[+t7.7] w[+mC]=UAS-wg[2x]-intergenic[2x].pCFD6}attP40 / P{y[+t7.7] w[+mC]=UAS-Cas9.P2}attP40, P{w[+mC]=tubP-GAL80[ts]}10] ; P{w[+mC]=hh-Gal4} / P{w[+mC]=UAS-p53.H159N.Ex}3* |
|  | C; p53 RNAi | hh-Gal4, tubGal80[ts] > Cas9.P2, wg+intergenic sgRNA + p53 RNAi | *w ; P{y[+t7.7] w[+mC]=UAS-wg[2x]-intergenic[2x].pCFD6}attP40 / P{y[+t7.7] w[+mC]=UAS-Cas9.P2}attP40, P{w[+mC]=tubP-GAL80[ts]}10] ; P{w[+mC]=hh-Gal4} / P{y[+t7.7] v[+t1.8]=TRiP.JF02513}attP2* |
|  | C; E2F RNAi | hh-Gal4, tubGal80[ts] > Cas9.P2, wg+intergenic sgRNA + E2F RNAi | *w ; P{y[+t7.7] w[+mC]=UAS-wg[2x]-intergenic[2x].pCFD6}attP40 / P{y[+t7.7] w[+mC]=UAS-Cas9.P2}attP40, P{w[+mC]=tubP-GAL80[ts]}10] ; P{w[+mC]=hh-Gal4} / P{y[+t7.7] v[+t1.8]=TRiP.JF02513}attP2* |
|  | C; UAS:Rbf | hh-Gal4, tubGal80[ts] > Cas9.P2, wg+intergenic sgRNA + UAS-Rbf | *w ; P{y[+t7.7] w[+mC]=UAS-wg[2x]-intergenic[2x].pCFD6}attP40 / P{y[+t7.7] w[+mC]=UAS-Cas9.P2}attP40, P{w[+mC]=tubP-GAL80[ts]}10] ; P{w[+mC]=hh-Gal4} / P{w[+mC]=UAS-Rbf.D}III* |
|  | D; EGFRgammatop | hh-Gal4, tubGal80[ts] > Cas9.P2, wg+intergenic sgRNA + UAS:EGFR-gammatop | *w ; P{y[+t7.7] w[+mC]=UAS-wg[2x]-intergenic[2x].pCFD6}attP40 / P{y[+t7.7] w[+mC]=UAS-Cas9.P2}attP40, P{w[+mC]=tubP-GAL80[ts]}10] ; P{w[+mC]=hh-Gal4} / P{w[+mC]=UAS-Egfr.lambdatop}3* |
|  | D; UAS-vn | hh-Gal4, tubGal80[ts] > Cas9.P2, wg+intergenic sgRNA + UAS-vn | *UAS-vn ; P{y[+t7.7] w[+mC]=UAS-wg[2x]-intergenic[2x].pCFD6}attP40 / P{y[+t7.7] w[+mC]=UAS-Cas9.P2}attP40, P{w[+mC]=tubP-GAL80[ts]}10] ; P{w[+mC]=hh-Gal4}* |
|  | D; UAS-krn | hh-Gal4, tubGal80[ts] > Cas9.P2, wg+intergenic sgRNA + UAS-s-krn | *w ; P{y[+t7.7] w[+mC]=UAS-wg[2x]-intergenic[2x].pCFD6}attP40 / P{y[+t7.7] w[+mC]=UAS-Cas9.P2}attP40, P{w[+mC]=tubP-GAL80[ts]}10] ; P{w[+mC]=hh-Gal4} / UAS-s-Krn* |
|  | D; UAS-grk | hh-Gal4, tubGal80[ts] > Cas9.P2, wg+intergenic sgRNA + UAS-s-grk | *w ; P{y[+t7.7] w[+mC]=UAS-wg[2x]-intergenic[2x].pCFD6}attP40 / P{y[+t7.7] w[+mC]=UAS-Cas9.P2}attP40, P{w[+mC]=tubP-GAL80[ts]}10] ; P{w[+mC]=hh-Gal4} / UAS-s-grk* |
|  |  |  |  |
| Figure 4 | A, left | hh-Gal4, tubGal80[ts] > Cas9.P2, wg+intergenic sgRNA | *w ; P{y[+t7.7] w[+mC]=UAS-wg[2x]-intergenic[2x].pCFD6}attP40 / P{y[+t7.7] w[+mC]=UAS-Cas9.P2}attP40, P{w[+mC]=tubP-GAL80[ts]}10] ; P{w[+mC]=hh-Gal4}* |
|  | A, right | hh-Gal4, tubGal80[ts] > Cas9.P2, wg+intergenic sgRNA + hid RNAi | *w ; P{y[+t7.7] w[+mC]=UAS-wg[2x]-intergenic[2x].pCFD6}attP40 / P{y[+t7.7] w[+mC]=UAS-Cas9.P2}attP40, P{w[+mC]=tubP-GAL80[ts]}10] ; P{w[+mC]=hh-Gal4} / P{GD8269}* |
|  | C, left | hh-Gal4, tubGal80[ts] > Cas9.P2, hid-EGFP | *w ; P{y[+t7.7] w[+mC]=UAS-Cas9.P2}attP40, P{w[+mC]=tubP-GAL80[ts]}10] ; P{w[+mC]=hh-Gal4} / P{w[+mC]=hid-EGFP.5'F-WT}3* |
|  | C, middle | hh-Gal4, tubGal80[ts] > Cas9.P2, hid-EGFP, intergenic sgRNA | *w ; P{y[+t7.7] w[+mC]=UAS-Cas9.P2}attP40, P{w[+mC]=tubP-GAL80[ts]}10] / P{y[+t7.7] w[+mC]=UAS-intergenic[2x].pCFD6}attP40 ; P{w[+mC]=hh-Gal4} / P{w[+mC]=hid-EGFP.5'F-WT}3* |
|  | C, right | hh-Gal4, tubGal80[ts] > Cas9.P2, hid-EGFP, wg+intergenic sgRNA | *w ; P{y[+t7.7] w[+mC]=UAS-Cas9.P2}attP40, P{w[+mC]=tubP-GAL80[ts]}10] / P{y[+t7.7] w[+mC]=UAS-wg[2x]-intergenic[2x].pCFD6}attP40 ; P{w[+mC]=hh-Gal4} / P{w[+mC]=hid-EGFP.5'F-WT}3* |
|  |  |  |  |
| Figure 5 | A; left (top) | hh-Gal4, tubGal80[ts] > mCherry RNAi | *w / y[1] sc[*] v[1] sev[21] ; P{w[+mC]=tubP-GAL80[ts]}10]; P{y[+t7.7] v[+t1.8]=VALIUM20-mCherry.RNAi}attP2 / P{w[+mC]=hh-Gal4}* |
|  | A; left (bottom) | hh-Gal4, tubGal80[ts] > w | *w ; P{w[+mC]=tubP-GAL80[ts]}10] / P{w[+mC]=hh-Gal4}* |
|  | A; second from left | hh-Gal4, tubGal80[ts] > wg RNAi | *w / y[1] sc[*] v[1] sev[21] ; P{w[+mC]=tubP-GAL80[ts]}10]; P{y[+t7.7] v[+t1.8]=TRiP.HMS00794}attP2 / P{w[+mC]=hh-Gal4}* |
|  | A; third from left | hh-Gal4, tubGal80[ts] > arm RNAi | *w / y[1] sc[*] v[1] sev[21] ; P{w[+mC]=tubP-GAL80[ts]}10]; P{y[+t7.7] v[+t1.8]=TRiP.HMS01414}attP2 / P{w[+mC]=hh-Gal4}* |
|  | A; right | hh-Gal4, tubGal80[ts] > UAS:wg | *w ; P{w[+mC]=tubP-GAL80[ts]}10]; P{w[+mC]=UAS-wg.H.T:HA1}6C / P{w[+mC]=hh-Gal4}* |
|  | B; left | hh-Gal4, tubGal80[ts] > UAS:wg | *w; P{w[+mC]=tubP-GAL80[ts]}10]; P{w[+mC]=UAS-wg.H.T:HA1}6C / P{w[+mC]=hh-Gal4}* |
|  | B; right | hh-Gal4, tubGal80[ts] > UAS:wg, rho RNAi | *w / y[1] sc[*] v[1] sev[21] ; P{w[+mC]=tubP-GAL80[ts]}10]; P{w[+mC]=UAS-wg.H.T:HA1}6C, P{y[+t7.7] v[+t1.8]=TRiP.JF03106}attP2 / P{w[+mC]=hh-Gal4}* |
|  |  |  |  |
| Figure 6 | A, left | hh-Gal4, tubGal80[ts] > Cas9.P2, wg+intergenic sgRNA | *w ; P{y[+t7.7] w[+mC]=UAS-wg[2x]-intergenic[2x].pCFD6}attP40 / P{y[+t7.7] w[+mC]=UAS-Cas9.P2}attP40, P{w[+mC]=tubP-GAL80[ts]}10] ; P{w[+mC]=hh-Gal4}* |
|  | A, right | hh-Gal4, tubGal80[ts] > Cas9.P2, wg+intergenic sgRNA, UAS:rho | *w ; P{y[+t7.7] w[+mC]=UAS-wg[2x]-intergenic[2x].pCFD6}attP40 / P{y[+t7.7] w[+mC]=UAS-Cas9.P2}attP40, P{w[+mC]=tubP-GAL80[ts]}10] ; P{w[+mC]=hh-Gal4} / UAS-rhomboid* |
|  | C, left | hh-Gal4, tubGal80[ts] > Cas9.P2, intergenic sgRNA | *w ; P{y[+t7.7] w[+mC]=UAS-intergenic[2x].pCFD6}attP40 / P{y[+t7.7] w[+mC]=UAS-Cas9.P2}attP40, P{w[+mC]=tubP-GAL80[ts]}10] ; P{w[+mC]=hh-Gal4}* |
|  | C, second from left | hh-Gal4, tubGal80[ts] > Cas9.P2, intergenic sgRNA, erk RNAi | *w / y[1] sc[*] v[1] sev[21] ; P{y[+t7.7] w[+mC]=UAS-intergenic[2x].pCFD6}attP40 / P{y[+t7.7] w[+mC]=UAS-Cas9.P2}attP40, P{w[+mC]=tubP-GAL80[ts]}10] ; P{w[+mC]=hh-Gal4} / P{y[+t7.7] v[+t1.8]=TRiP.HMS00173}attP2* |
|  | C, third from left | hh-Gal4, tubGal80[ts] > Cas9.P2, non-targeting sgRNA, erk RNAi | *w / y[1] sc[*] v[1] sev[21] ; P{y[+t7.7] w[+mC]=UAS-QUAS[2x].pCFD6}attP40 / P{y[+t7.7] w[+mC]=UAS-Cas9.P2}attP40, P{w[+mC]=tubP-GAL80[ts]}10] ; P{w[+mC]=hh-Gal4} / P{y[+t7.7] v[+t1.8]=TRiP.HMS00173}attP2* |
|  | C, fourth from left | hh-Gal4, tubGal80[ts] > Cas9.P2, intergenic sgRNA, rho RNAi | *w / y[1] v[1] ; P{y[+t7.7] w[+mC]=UAS-intergenic[2x].pCFD6}attP40 / P{y[+t7.7] w[+mC]=UAS-Cas9.P2}attP40, P{w[+mC]=tubP-GAL80[ts]}10] ; P{w[+mC]=hh-Gal4} / P{y[+t7.7] v[+t1.8]=TRiP.JF03106}attP2* |
|  | C, right | hh-Gal4, tubGal80[ts] > Cas9.P2, non-targeting sgRNA, rho RNAi | *w / y[1] v[1] ; P{y[+t7.7] w[+mC]=UAS-QUAS[2x].pCFD6}attP40 / P{y[+t7.7] w[+mC]=UAS-Cas9.P2}attP40, P{w[+mC]=tubP-GAL80[ts]}10] ; P{w[+mC]=hh-Gal4} / P{y[+t7.7] v[+t1.8]=TRiP.JF03106}attP2* |
|  | E, left | hh-Gal4, tubGal80[ts] > Cas9.P2, non-targeting sgRNA | *w ; P{y[+t7.7] w[+mC]=UAS-QUAS[2x].pCFD6}attP40 / P{y[+t7.7] w[+mC]=UAS-Cas9.P2}attP40, P{w[+mC]=tubP-GAL80[ts]}10] ; P{w[+mC]=hh-Gal4}* |
|  | E, second from left | hh-Gal4, tubGal80[ts] > Cas9.P2, intergenic sgRNA | *w ; P{y[+t7.7] w[+mC]=UAS-intergenic[2x].pCFD6}attP40 / P{y[+t7.7] w[+mC]=UAS-Cas9.P2}attP40, P{w[+mC]=tubP-GAL80[ts]}10] ; P{w[+mC]=hh-Gal4}* |
|  | E, third from left | hh-Gal4, tubGal80[ts] > Cas9.P2, UAS-wg, non-targeting sgRNA | *w ; P{y[+t7.7] w[+mC]=UAS-QUAS[2x].pCFD6}attP40 / P{y[+t7.7] w[+mC]=UAS-Cas9.P2}attP40, P{w[+mC]=tubP-GAL80[ts]}10] ; P{w[+mC]=hh-Gal4} / P{w[+mC]=UAS-wg.H.T:HA1}6C* |
|  | E, fourth from left | hh-Gal4, tubGal80[ts] > Cas9.P2, UAS-wg, intergenic sgRNA | *w ; P{y[+t7.7] w[+mC]=UAS-intergenic[2x].pCFD6}attP40 / P{y[+t7.7] w[+mC]=UAS-Cas9.P2}attP40, P{w[+mC]=tubP-GAL80[ts]}10] ; P{w[+mC]=hh-Gal4} / P{w[+mC]=UAS-wg.H.T:HA1}6C* |
|  | E, fifth from left | hh-Gal4, tubGal80[ts] > Cas9.P2, UAS-wg + erk RNAi, non-targeting sgRNA | *w ; P{y[+t7.7] w[+mC]=UAS-QUAS[2x].pCFD6}attP40 / P{y[+t7.7] w[+mC]=UAS-Cas9.P2}attP40, P{w[+mC]=tubP-GAL80[ts]}10] ; P{w[+mC]=hh-Gal4} / P{w[+mC]=UAS-wg.H.T:HA1}6C, P{y[+t7.7] v[+t1.8]=TRiP.HMS00173}attP2* |
|  | E, sixth from left | hh-Gal4, tubGal80[ts] > Cas9.P2, UAS-wg + erk RNAi, intergenic sgRNA | *w ; P{y[+t7.7] w[+mC]=UAS-intergenic[2x].pCFD6}attP40 / P{y[+t7.7] w[+mC]=UAS-Cas9.P2}attP40, P{w[+mC]=tubP-GAL80[ts]}10] ; P{w[+mC]=hh-Gal4} / P{w[+mC]=UAS-wg.H.T:HA1}6C, P{y[+t7.7] v[+t1.8]=TRiP.HMS00173}attP2* |
|  | E, seventh from left | hh-Gal4, tubGal80[ts] > Cas9.P2, UAS-wg + rho RNAi, non-targeting sgRNA | *w ; P{y[+t7.7] w[+mC]=UAS-QUAS[2x].pCFD6}attP40 / P{y[+t7.7] w[+mC]=UAS-Cas9.P2}attP40, P{w[+mC]=tubP-GAL80[ts]}10] ; P{w[+mC]=hh-Gal4} / P{w[+mC]=UAS-wg.H.T:HA1}6C, P{y[+t7.7] v[+t1.8]=TRiP.JF03106}attP2* |
|  | E, right | hh-Gal4, tubGal80[ts] > Cas9.P2, UAS-wg + rho RNAi, intergenic sgRNA | *w ; P{y[+t7.7] w[+mC]=UAS-intergenic[2x].pCFD6}attP40 / P{y[+t7.7] w[+mC]=UAS-Cas9.P2}attP40, P{w[+mC]=tubP-GAL80[ts]}10] ; P{w[+mC]=hh-Gal4} / P{w[+mC]=UAS-wg.H.T:HA1}6C, P{y[+t7.7] v[+t1.8]=TRiP.JF03106}attP2* |
|  |  |  |  |
| Figure S1 | A | hh-Gal4 > Cas9.P2, wg sgRNA | *w ; P{y[+t7.7] w[+mC]=UAS-wg[2x].pCFD6}attP40 / P{y[+t7.7] w[+mC]=UAS-Cas9.P2}attP40 ; P{w[+mC]=hh-Gal4}* |
|  |  | hh-Gal4 > Cas9.P2, wnt2 sgRNA | *w ; P{y[+t7.7] w[+mC]=UAS-wnt2[2x].pCFD6}attP40 / P{y[+t7.7] w[+mC]=UAS-Cas9.P2}attP40 ; P{w[+mC]=hh-Gal4}* |
|  |  | hh-Gal4 > Cas9.P2, wnt4 sgRNA | *w ; P{y[+t7.7] w[+mC]=UAS-wnt4[2x].pCFD6}attP40 / P{y[+t7.7] w[+mC]=UAS-Cas9.P2}attP40 ; P{w[+mC]=hh-Gal4}* |
|  |  | hh-Gal4 > Cas9.P2, wnt5 sgRNA | *w ; P{y[+t7.7] w[+mC]=UAS-wnt5[2x].pCFD6}attP40 / P{y[+t7.7] w[+mC]=UAS-Cas9.P2}attP40 ; P{w[+mC]=hh-Gal4}* |
|  |  | hh-Gal4 > Cas9.P2, wnt6 sgRNA | *w ; P{y[+t7.7] w[+mC]=UAS-wnt6[2x].pCFD6}attP40 / P{y[+t7.7] w[+mC]=UAS-Cas9.P2}attP40 ; P{w[+mC]=hh-Gal4}* |
|  |  | hh-Gal4 > Cas9.P2, wntD sgRNA | *w ; P{y[+t7.7] w[+mC]=UAS-wntD[2x].pCFD6}attP40 / P{y[+t7.7] w[+mC]=UAS-Cas9.P2}attP40 ; P{w[+mC]=hh-Gal4}* |
|  |  | hh-Gal4 > Cas9.P2, wnt10 sgRNA | *w ; P{y[+t7.7] w[+mC]=UAS-wnt10[2x].pCFD6}attP40 / P{y[+t7.7] w[+mC]=UAS-Cas9.P2}attP40 ; P{w[+mC]=hh-Gal4}* |
|  |  | hh-Gal4 > Cas9.P2, wg+wnt2 sgRNA | *w ; P{y[+t7.7] w[+mC]=UAS-wg[2x]-wnt2[2x].pCFD6}attP40 / P{y[+t7.7] w[+mC]=UAS-Cas9.P2}attP40 ; P{w[+mC]=hh-Gal4}* |
|  |  | hh-Gal4 > Cas9.P2, wg+wnt4 sgRNA | *w ; P{y[+t7.7] w[+mC]=UAS-wg[2x]-wnt4[2x].pCFD6}attP40 / P{y[+t7.7] w[+mC]=UAS-Cas9.P2}attP40 ; P{w[+mC]=hh-Gal4}* |
|  |  | hh-Gal4 > Cas9.P2, wg+wnt5 sgRNA | *w ; P{y[+t7.7] w[+mC]=UAS-wg[2x]-wnt5[2x].pCFD6}attP40 / P{y[+t7.7] w[+mC]=UAS-Cas9.P2}attP40 ; P{w[+mC]=hh-Gal4}* |
|  |  | hh-Gal4 > Cas9.P2, wg+wnt6 sgRNA | *w ; P{y[+t7.7] w[+mC]=UAS-wg[2x]-wnt6[2x].pCFD6}attP40 / P{y[+t7.7] w[+mC]=UAS-Cas9.P2}attP40 ; P{w[+mC]=hh-Gal4}* |
|  |  | hh-Gal4 > Cas9.P2, wg+wntD sgRNA | *w ; P{y[+t7.7] w[+mC]=UAS-wg[2x]-wntD[2x].pCFD6}attP40 / P{y[+t7.7] w[+mC]=UAS-Cas9.P2}attP40 ; P{w[+mC]=hh-Gal4}* |
|  |  | hh-Gal4 > Cas9.P2, wg+wnt10 sgRNA | *w ; P{y[+t7.7] w[+mC]=UAS-wg[2x]-wnt10[2x].pCFD6}attP40 / P{y[+t7.7] w[+mC]=UAS-Cas9.P2}attP40 ; P{w[+mC]=hh-Gal4}* |
|  |  | hh-Gal4 > Cas9.P2, wnt2+wnt4 sgRNA | *w ; P{y[+t7.7] w[+mC]=UAS-wnt2[2x]-wnt4[2x].pCFD6}attP40 / P{y[+t7.7] w[+mC]=UAS-Cas9.P2}attP40 ; P{w[+mC]=hh-Gal4}* |
|  |  | hh-Gal4 > Cas9.P2, wnt2+wnt5 sgRNA | *w ; P{y[+t7.7] w[+mC]=UAS-wnt2[2x]-wnt5[2x].pCFD6}attP40 / P{y[+t7.7] w[+mC]=UAS-Cas9.P2}attP40 ; P{w[+mC]=hh-Gal4}* |
|  |  | hh-Gal4 > Cas9.P2, wnt2+wnt6 sgRNA | *w ; P{y[+t7.7] w[+mC]=UAS-wnt2[2x]-wnt6[2x].pCFD6}attP40 / P{y[+t7.7] w[+mC]=UAS-Cas9.P2}attP40 ; P{w[+mC]=hh-Gal4}* |
|  |  | hh-Gal4 > Cas9.P2, wnt2+wntD sgRNA | *w ; P{y[+t7.7] w[+mC]=UAS-wnt2[2x]-wntD[2x].pCFD6}attP40 / P{y[+t7.7] w[+mC]=UAS-Cas9.P2}attP40 ; P{w[+mC]=hh-Gal4}* |
|  |  | hh-Gal4 > Cas9.P2, wnt2+wnt10 sgRNA | *w ; P{y[+t7.7] w[+mC]=UAS-wnt2[2x]-wnt10[2x].pCFD6}attP40 / P{y[+t7.7] w[+mC]=UAS-Cas9.P2}attP40 ; P{w[+mC]=hh-Gal4}* |
|  |  | hh-Gal4 > Cas9.P2, wnt4+wnt5 sgRNA | *w ; P{y[+t7.7] w[+mC]=UAS-wnt4[2x]-wnt5[2x].pCFD6}attP40 / P{y[+t7.7] w[+mC]=UAS-Cas9.P2}attP40 ; P{w[+mC]=hh-Gal4}* |
|  |  | hh-Gal4 > Cas9.P2, wnt4+wnt6 sgRNA | *w ; P{y[+t7.7] w[+mC]=UAS-wnt4[2x]-wnt6[2x].pCFD6}attP40 / P{y[+t7.7] w[+mC]=UAS-Cas9.P2}attP40 ; P{w[+mC]=hh-Gal4}* |
|  |  | hh-Gal4 > Cas9.P2, wnt4+wntD sgRNA | *w ; P{y[+t7.7] w[+mC]=UAS-wnt4[2x]-wntD[2x].pCFD6}attP40 / P{y[+t7.7] w[+mC]=UAS-Cas9.P2}attP40 ; P{w[+mC]=hh-Gal4}* |
|  |  | hh-Gal4 > Cas9.P2, wnt4+wnt10 sgRNA | *w ; P{y[+t7.7] w[+mC]=UAS-wnt4[2x]-wnt10[2x].pCFD6}attP40 / P{y[+t7.7] w[+mC]=UAS-Cas9.P2}attP40 ; P{w[+mC]=hh-Gal4}* |
|  |  | hh-Gal4 > Cas9.P2, wnt5+wnt6 sgRNA | *w ; P{y[+t7.7] w[+mC]=UAS-wnt5[2x]-wnt6[2x].pCFD6}attP40 / P{y[+t7.7] w[+mC]=UAS-Cas9.P2}attP40 ; P{w[+mC]=hh-Gal4}* |
|  |  | hh-Gal4 > Cas9.P2, wnt5+wntD sgRNA | *w ; P{y[+t7.7] w[+mC]=UAS-wnt5[2x]-wntD[2x].pCFD6}attP40 / P{y[+t7.7] w[+mC]=UAS-Cas9.P2}attP40 ; P{w[+mC]=hh-Gal4}* |
|  |  | hh-Gal4 > Cas9.P2, wnt5+wnt10 sgRNA | *w ; P{y[+t7.7] w[+mC]=UAS-wnt5[2x]-wnt10[2x].pCFD6}attP40 / P{y[+t7.7] w[+mC]=UAS-Cas9.P2}attP40 ; P{w[+mC]=hh-Gal4}* |
|  |  | hh-Gal4 > Cas9.P2, wnt6+wntD sgRNA | *w ; P{y[+t7.7] w[+mC]=UAS-wnt6[2x]-wntD[2x].pCFD6}attP40 / P{y[+t7.7] w[+mC]=UAS-Cas9.P2}attP40 ; P{w[+mC]=hh-Gal4}* |
|  |  | hh-Gal4 > Cas9.P2, wnt6+wnt10 sgRNA | *w ; P{y[+t7.7] w[+mC]=UAS-wnt6[2x]-wnt10[2x].pCFD6}attP40 / P{y[+t7.7] w[+mC]=UAS-Cas9.P2}attP40 ; P{w[+mC]=hh-Gal4}* |
|  |  | hh-Gal4 > Cas9.P2, wntD+wnt10 sgRNA | *w ; P{y[+t7.7] w[+mC]=UAS-wntD[2x]-wnt10[2x].pCFD6}attP40 / P{y[+t7.7] w[+mC]=UAS-Cas9.P2}attP40 ; P{w[+mC]=hh-Gal4}* |
|  |  | hh-Gal4 > Cas9.P2, intergenic sgRNA | *w ; P{y[+t7.7] w[+mC]=UAS-intergenic[2x].pCFD6}attP40 / P{y[+t7.7] w[+mC]=UAS-Cas9.P2}attP40 ; P{w[+mC]=hh-Gal4}* |
|  |  | hh-Gal4 > Cas9.P2, wg+intergenic sgRNA | *w ; P{y[+t7.7] w[+mC]=UAS-wg[2x]-intergenic[2x].pCFD6}attP40 / P{y[+t7.7] w[+mC]=UAS-Cas9.P2}attP40 ; P{w[+mC]=hh-Gal4}* |
|  |  | hh-Gal4 > Cas9.P2, wg+intergenic-2 sgRNA | *w ; P{y[+t7.7] w[+mC]=UAS-wg[2x]-intergenic-2[2x].pCFD6}attP40 / P{y[+t7.7] w[+mC]=UAS-Cas9.P2}attP40 ; P{w[+mC]=hh-Gal4}* |
|  |  | hh-Gal4 > Cas9.P2, wnt2+intergenic | *w ; P{y[+t7.7] w[+mC]=UAS-wnt2[2x]-intergenic[2x].pCFD6}attP40 / P{y[+t7.7] w[+mC]=UAS-Cas9.P2}attP40 ; P{w[+mC]=hh-Gal4}* |
|  |  | hh-Gal4 > Cas9.P2, wnt10+intergenic | *w ; P{y[+t7.7] w[+mC]=UAS-wnt10[2x]-intergenic[2x].pCFD6}attP40 / P{y[+t7.7] w[+mC]=UAS-Cas9.P2}attP40 ; P{w[+mC]=hh-Gal4}* |
|  |  | hh-Gal4 > Cas9.P2, wntless/evi RNAi | *w ; P{y[+t7.7] w[+mC]=UAS-evi-pFP854.pCFD6}attP40 ; P{w[+mC]=hh-Gal4}* |
|  |  |  |  |
| Figure S2 |  | hh-Gal4 > Cas9.P2, no sgRNA | *w ; P{y[+t7.7] w[+mC]=UAS-Cas9.P2}attP40 ; P{w[+mC]=hh-Gal4}* |
|  |  | hh-Gal4 > Cas9.P2, non-targeting sgRNA | *w ; P{y[+t7.7] w[+mC]=UAS-QUAS[2x].pCFD6}attP40 / P{y[+t7.7] w[+mC]=UAS-Cas9.P2}attP40 ; P{w[+mC]=hh-Gal4}* |
|  |  | hh-Gal4 > Cas9.P2, intergenic sgRNA | *w ; P{y[+t7.7] w[+mC]=UAS-intergenic[2x].pCFD6}attP40 / P{y[+t7.7] w[+mC]=UAS-Cas9.P2}attP40 ; P{w[+mC]=hh-Gal4}* |
|  |  | hh-Gal4 > Cas9.P2, wnt6 sgRNA | *w ; P{y[+t7.7] w[+mC]=UAS-wnt6[2x].pCFD6}attP40 / P{y[+t7.7] w[+mC]=UAS-Cas9.P2}attP40 ; P{w[+mC]=hh-Gal4}* |
|  |  | hh-Gal4 > Cas9.P2, wg sgRNA | *w ; P{y[+t7.7] w[+mC]=UAS-wg[2x].pCFD6}attP40 / P{y[+t7.7] w[+mC]=UAS-Cas9.P2}attP40 ; P{w[+mC]=hh-Gal4}* |
|  |  | hh-Gal4 > Cas9.P2, wg+wnt6 sgRNA | *w ; P{y[+t7.7] w[+mC]=UAS-wg[2x]-wnt6[2x].pCFD6}attP40 / P{y[+t7.7] w[+mC]=UAS-Cas9.P2}attP40 ; P{w[+mC]=hh-Gal4}* |
|  |  | hh-Gal4 > Cas9.P2, wnt4+wnt6 sgRNA | *w ; P{y[+t7.7] w[+mC]=UAS-wnt4[2x]-wnt6[2x].pCFD6}attP40 / P{y[+t7.7] w[+mC]=UAS-Cas9.P2}attP40 ; P{w[+mC]=hh-Gal4}* |
|  |  | hh-Gal4 > Cas9.P2, wnt5+wnt6 sgRNA | *w ; P{y[+t7.7] w[+mC]=UAS-wnt5[2x]-wnt6[2x].pCFD6}attP40 / P{y[+t7.7] w[+mC]=UAS-Cas9.P2}attP40 ; P{w[+mC]=hh-Gal4}* |
|  |  |  |  |
| Figure S3 | A, left | nub-Gal4, tubGal80[ts] > Cas9.P2, mCherry RNAi + non-targeting sgRNA | *w ; P{y[+t7.7] w[+mC]=UAS-QUAS[2x].pCFD6}attP40 / P{w[+mW.hs]=GawB}nubbin-AC-62; P{y[+t7.7] v[+t1.8]=VALIUM20-mCherry.RNAi}attP2 / P{y[+t7.7] w[+mC]=UAS-Cas9.P2}attP2, P{w[+mC]=tubP-GAL80[ts]}2* |
|  | A, second from left | nub-Gal4, tubGal80[ts] > Cas9.P2, mCherry RNAi + intergenic sgRNA | *w ; P{y[+t7.7] w[+mC]=UAS-intergenic[2x].pCFD6}attP40 / P{w[+mW.hs]=GawB}nubbin-AC-62; P{y[+t7.7] v[+t1.8]=VALIUM20-mCherry.RNAi}attP2 / P{y[+t7.7] w[+mC]=UAS-Cas9.P2}attP2, P{w[+mC]=tubP-GAL80[ts]}2* |
|  | A, third from left | nub-Gal4, tubGal80[ts] > Cas9.P2, wg RNAi + non-targeting sgRNA | *w ; P{y[+t7.7] w[+mC]=UAS-QUAS[2x].pCFD6}attP40 / P{w[+mW.hs]=GawB}nubbin-AC-62; P{y[+t7.7] v[+t1.8]=TRiP.HMS00794}attP2 / P{y[+t7.7] w[+mC]=UAS-Cas9.P2}attP2, P{w[+mC]=tubP-GAL80[ts]}2* |
|  | A, fourth from left | nub-Gal4, tubGal80[ts] > Cas9.P2, wg RNAi + intergenic sgRNA | *w ; P{y[+t7.7] w[+mC]=UAS-intergenic[2x].pCFD6}attP40 / P{w[+mW.hs]=GawB}nubbin-AC-62; P{y[+t7.7] v[+t1.8]=TRiP.HMS00794}attP2 / P{y[+t7.7] w[+mC]=UAS-Cas9.P2}attP2, P{w[+mC]=tubP-GAL80[ts]}2* |
|  | A, fifth from left | nub-Gal4, tubGal80[ts] > Cas9.P2, UAS-wg + non-targeting sgRNA | *w ; P{y[+t7.7] w[+mC]=UAS-QUAS[2x].pCFD6}attP40 / P{w[+mW.hs]=GawB}nubbin-AC-62; P{w[+mC]=UAS-wg.H.T:HA1}6C / P{y[+t7.7] w[+mC]=UAS-Cas9.P2}attP2, P{w[+mC]=tubP-GAL80[ts]}2* |
|  | A, right | nub-Gal4, tubGal80[ts] > Cas9.P2, UAS-wg + intergenic sgRNA | *w ; P{y[+t7.7] w[+mC]=UAS-intergenic[2x].pCFD6}attP40 / P{w[+mW.hs]=GawB}nubbin-AC-62; P{w[+mC]=UAS-wg.H.T:HA1}6C / P{y[+t7.7] w[+mC]=UAS-Cas9.P2}attP2, P{w[+mC]=tubP-GAL80[ts]}2* |
|  | B, left | hh-Gal4 > UAS:Cas9.P2, mCherry RNAi + intergenic sgRNA | *w ; P{y[+t7.7] w[+mC]=UAS-intergenic[2x].pCFD6}attP40 / P{y[+t7.7] w[+mC]=UAS-Cas9.P2}attP40 ; P{y[+t7.7] v[+t1.8]=VALIUM20-mCherry.RNAi}attP2 / P{w[+mC]=hh-Gal4}* |
|  | B, middle | hh-Gal4 > UAS:Cas9.P2, wg RNAi + intergenic sgRNA | *w ; P{y[+t7.7] w[+mC]=UAS-intergenic[2x].pCFD6}attP40 / P{y[+t7.7] w[+mC]=UAS-Cas9.P2}attP40 ; P{y[+t7.7] v[+t1.8]=TRiP.HMS00794}attP2 / P{w[+mC]=hh-Gal4}* |
|  | B, right | hh-Gal4 > UAS:Cas9.P2, wg RNAi + non-targeting sgRNA | *w ; P{y[+t7.7] w[+mC]=UAS-QUAS[2x].pCFD6}attP40 / P{y[+t7.7] w[+mC]=UAS-Cas9.P2}attP40 ; P{y[+t7.7] v[+t1.8]=TRiP.HMS00794}attP2 / P{w[+mC]=hh-Gal4}* |
|  | C, left | hh-Gal4 > UAS:Cas9.P2, mCherry RNAi + yellow sgRNA | *w ; P{y[+t7.7] w[+mC]=UAS-yellow-pFB407.pCFD6}attP40 / P{y[+t7.7] w[+mC]=UAS-Cas9.P2}attP40 ; P{y[+t7.7] v[+t1.8]=VALIUM20-mCherry.RNAi}attP2 / P{w[+mC]=hh-Gal4}* |
|  | C, right | hh-Gal4 > UAS:Cas9.P2, wg RNAi + yellow sgRNA | *w ; P{y[+t7.7] w[+mC]=UAS-yellow-pFB407.pCFD6}attP40/ P{y[+t7.7] w[+mC]=UAS-Cas9.P2}attP40 ; P{y[+t7.7] v[+t1.8]=TRiP.HMS00794}attP2 / P{w[+mC]=hh-Gal4}* |
|  | D, left | hh-Gal4 > UAS:u[M]-Cas9, mCherry RNAi + intergenic sgRNA | *w ; P{y[+t7.7] w[+mC]=UAS-intergenic[2x].pCFD6}attP40 / P{y[+t7.7] w[+mC]=UAS-uMCas9}attP40 ; P{y[+t7.7] v[+t1.8]=VALIUM20-mCherry.RNAi}attP2 / P{w[+mC]=hh-Gal4}* |
|  | D, middle | hh-Gal4 > UAS:u[M]-Cas9, wg RNAi + intergenic sgRNA | *w ; P{y[+t7.7] w[+mC]=UAS-intergenic[2x].pCFD6}attP40 / P{y[+t7.7] w[+mC]=UAS-uMCas9}attP40 ; P{y[+t7.7] v[+t1.8]=TRiP.HMS00794}attP2 / P{w[+mC]=hh-Gal4}* |
|  | D, right | hh-Gal4 > UAS:u[M]-Cas9, wg RNAi + non-targeting sgRNA | *w ; P{y[+t7.7] w[+mC]=UAS-QUAS[2x].pCFD6}attP40 / P{y[+t7.7] w[+mC]=UAS-uMCas9}attP40 ; P{y[+t7.7] v[+t1.8]=TRiP.HMS00794}attP2 / P{w[+mC]=hh-Gal4}* |
|  | E, top left | nub-Gal4 > UAS:u[M]-Cas9, no sgRNA | *w ; P{w[+mW.hs]=GawB}nubbin-AC-62; P{w[+mC]=UAS-wg.H.T:HA1}6C / P{y[+t7.7] w[+mC]=UAS-uMCas9}attP40* |
|  | E, top middle | nub-Gal4 > UAS:u[M]-Cas9, non-targeting sgRNA | *w ; P{y[+t7.7] w[+mC]=UAS-QUAS[2x].pCFD6}attP40 / P{w[+mW.hs]=GawB}nubbin-AC-62; P{w[+mC]=UAS-wg.H.T:HA1}6C / P{y[+t7.7] w[+mC]=UAS-uMCas9}attP40* |
|  | E, top right | nub-Gal4 > UAS:u[M]-Cas9, intergenic sgRNA | *w ; P{y[+t7.7] w[+mC]=UAS-intergenic[2x].pCFD6}attP40 / P{w[+mW.hs]=GawB}nubbin-AC-62; P{w[+mC]=UAS-wg.H.T:HA1}6C / P{y[+t7.7] w[+mC]=UAS-uMCas9}attP40* |
|  | E, middle left | nub-Gal4 > UAS:u[M]-Cas9, wg+wnt6 sgRNA | *w ; P{y[+t7.7] w[+mC]=UAS-wg[2x]-wnt6[2x].pCFD6}attP40 / P{w[+mW.hs]=GawB}nubbin-AC-62; P{w[+mC]=UAS-wg.H.T:HA1}6C / P{y[+t7.7] w[+mC]=UAS-uMCas9}attP40* |
|  | E, middle middle | nub-Gal4 > UAS:u[M]-Cas9, ebony-pFP545 sgRNA | *w ; P{y[+t7.7] w[+mC]=ebony-pFB545.pCFD6}attP40 / P{w[+mW.hs]=GawB}nubbin-AC-62; P{w[+mC]=UAS-wg.H.T:HA1}6C / P{y[+t7.7] w[+mC]=UAS-uMCas9}attP40* |
|  | E, middle right | nub-Gal4 > UAS:u[M]-Cas9, ebony-pFP578 sgRNA | *w ; P{y[+t7.7] w[+mC]=ebony-pFB578.pCFD6}attP40 / P{w[+mW.hs]=GawB}nubbin-AC-62; P{w[+mC]=UAS-wg.H.T:HA1}6C / P{y[+t7.7] w[+mC]=UAS-uMCas9}attP40* |
|  | E, bottom left | nub-Gal4 > UAS:u[M]-Cas9, yellow-pFP407 sgRNA | *w ; P{y[+t7.7] w[+mC]=yellow-pFB407.pCFD6}attP40 / P{w[+mW.hs]=GawB}nubbin-AC-62; P{w[+mC]=UAS-wg.H.T:HA1}6C / P{y[+t7.7] w[+mC]=UAS-uMCas9}attP40* |
|  | E, bottom middle | nub-Gal4 > UAS:u[M]-Cas9, oskar sgRNA | *w ; P{y[+t7.7] w[+mC]=oskar.pCFD6}attP40 / P{w[+mW.hs]=GawB}nubbin-AC-62; P{w[+mC]=UAS-wg.H.T:HA1}6C / P{y[+t7.7] w[+mC]=UAS-uMCas9}attP40* |
|  |  |  |  |
| Figure S4 | top row | hh-Gal4 > mCherry RNAi | *w / y[1] sc[*] v[1] sev[21] ; P{y[+t7.7] v[+t1.8]=VALIUM20-mCherry.RNAi}attP2 / P{w[+mC]=hh-Gal4}* |
|  | second row | hh-Gal4 > wg RNAi | *w / y[1] sc[*] v[1] sev[21] ; P{y[+t7.7] v[+t1.8]=TRiP.HMS00794}attP2 / P{w[+mC]=hh-Gal4}* |
|  | third row | hh-Gal4, tubGal80[ts] > mCherry RNAi | *w / y[1] sc[*] v[1] sev[21] ; P{w[+mC]=tubP-GAL80[ts]}10]; P{y[+t7.7] v[+t1.8]=VALIUM20-mCherry.RNAi}attP2 / P{w[+mC]=hh-Gal4}* |
|  | fourth row | hh-Gal4, tubGal80[ts] > arm RNAi | *w / y[1] sc[*] v[1] sev[21] ; P{w[+mC]=tubP-GAL80[ts]}10]; P{y[+t7.7] v[+t1.8]=TRiP.HMS01414}attP2 / P{w[+mC]=hh-Gal4}* |
|  |  |  |  |
| Figure S5 | rows 1, 3, 5 | en-Gal4, tubGal80[ts], UAS:GFP > UAS:dCas9-VPR, non-targeting sgRNA | w[*]; P{w[+mW.hs]=en2.4-GAL4}e16E, P{w[+mC]=UAS-2xEGFP}AH2 / P{y[+t7.7] w[+mC]=UAS-QUAS.pCFD6}attP40 ; P{y[+t7.7] w[+mC]=UAS-3xFLAG.dCas9.VPR}attP2, P{w[+mC]=tubP-GAL80[ts]}2 |
|  | rows 2, 4, 6 | en-Gal4, tubGal80[ts], UAS:GFP > UAS:dCas9-VPR, wg sgRNA | w[*]; P{w[+mW.hs]=en2.4-GAL4}e16E, P{w[+mC]=UAS-2xEGFP}AH2 / P{y[+t7.7] v[+t1.8]=TOE.GS00125}attP40 ; P{y[+t7.7] w[+mC]=UAS-3xFLAG.dCas9.VPR}attP2, P{w[+mC]=tubP-GAL80[ts]}2 |
|  |  |  |  |
| Figure S6 | B, left | hh-Gal4, tubGal80[ts] > Cas9.P2, non-targeting sgRNA | *w ; P{y[+t7.7] w[+mC]=UAS-QUAS[2x].pCFD6}attP40 / P{y[+t7.7] w[+mC]=UAS-Cas9.P2}attP40, P{w[+mC]=tubP-GAL80[ts]}10] ; P{w[+mC]=hh-Gal4}* |
|  | B, right | hh-Gal4, tubGal80[ts] > Cas9.P2, wg+intergenic sgRNA | *w ; P{y[+t7.7] w[+mC]=UAS-wg[2x]-intergenic[2x].pCFD6}attP40 / P{y[+t7.7] w[+mC]=UAS-Cas9.P2}attP40, P{w[+mC]=tubP-GAL80[ts]}10] ; P{w[+mC]=hh-Gal4}* |
|  |  |  |  |
|  | C, left top | hh-Gal4, tubGal80[ts] > Cas9.P2, wg+intergenic sgRNA, Chk1 RNAi-2 | *w ; P{y[+t7.7] w[+mC]=UAS-wg[2x]-intergenic[2x].pCFD6}attP40 / P{y[+t7.7] w[+mC]=UAS-Cas9.P2}attP40, P{w[+mC]=tubP-GAL80[ts]}10] ; P{w[+mC]=hh-Gal4} / P{y[+t7.7] v[+t1.8]=TRiP.HMS01573}attP2* |
|  | C, left second row | hh-Gal4, tubGal80[ts] > Cas9.P2, wg+intergenic sgRNA, Chk1 RNAi-1 | *w ; P{y[+t7.7] w[+mC]=UAS-wg[2x]-intergenic[2x].pCFD6}attP40 / P{y[+t7.7] w[+mC]=UAS-Cas9.P2}attP40, P{w[+mC]=tubP-GAL80[ts]}10] ; P{w[+mC]=hh-Gal4} / P{y[+t7.7] v[+t1.8]=TRiP.JF02588}attP2* |
|  | C, left third row | hh-Gal4, tubGal80[ts] > Cas9.P2, wg+intergenic sgRNA, ATR/mei-41 RNAi-1 | *w ; P{y[+t7.7] w[+mC]=UAS-wg[2x]-intergenic[2x].pCFD6}attP40 / P{y[+t7.7] w[+mC]=UAS-Cas9.P2}attP40, P{w[+mC]=tubP-GAL80[ts]}10] ; P{w[+mC]=hh-Gal4} / P{y[+t7.7] v[+t1.8]=TRiP.GL00284}attP2* |
|  | C, left fourth row | hh-Gal4, tubGal80[ts] > Cas9.P2, wg+intergenic sgRNA, ATM/tefu RNAi-1 | *w ; P{y[+t7.7] w[+mC]=UAS-wg[2x]-intergenic[2x].pCFD6}attP40 / P{y[+t7.7] w[+mC]=UAS-Cas9.P2}attP40, P{w[+mC]=tubP-GAL80[ts]}10] ; P{w[+mC]=hh-Gal4} / P{y[+t7.7] v[+t1.8]=TRiP.GL00138}attP2* |
|  | C, left bottom | hh-Gal4, tubGal80[ts] > Cas9.P2, wg+intergenic sgRNA, Chk2/lok RNAi-1 | *w ; P{y[+t7.7] w[+mC]=UAS-wg[2x]-intergenic[2x].pCFD6}attP40 / P{y[+t7.7] w[+mC]=UAS-Cas9.P2}attP40, P{w[+mC]=tubP-GAL80[ts]}10] ; P{w[+mC]=hh-Gal4} / P{y[+t7.7] v[+t1.8]=TRiP.GL00020}attP2* |
|  | C, right top | hh-Gal4, tubGal80[ts] > Cas9.P2, wg+intergenic sgRNA, p53[DN] | *w ; P{y[+t7.7] w[+mC]=UAS-wg[2x]-intergenic[2x].pCFD6}attP40 / P{y[+t7.7] w[+mC]=UAS-Cas9.P2}attP40, P{w[+mC]=tubP-GAL80[ts]}10] ; P{w[+mC]=hh-Gal4} / P{w[+mC]=UAS-p53.H159N.Ex}3* |
|  | C, right second row | hh-Gal4, tubGal80[ts] > Cas9.P2, wg+intergenic sgRNA, p53 RNAi | *w ; P{y[+t7.7] w[+mC]=UAS-wg[2x]-intergenic[2x].pCFD6}attP40 / P{y[+t7.7] w[+mC]=UAS-Cas9.P2}attP40, P{w[+mC]=tubP-GAL80[ts]}10] ; P{w[+mC]=hh-Gal4} / P{y[+t7.7] v[+t1.8]=TRiP.JF02513}attP2* |
|  | C, right third row | hh-Gal4, tubGal80[ts] > Cas9.P2, wg+intergenic sgRNA, E2F1 RNAi | *w ; P{y[+t7.7] w[+mC]=UAS-wg[2x]-intergenic[2x].pCFD6}attP40 / P{y[+t7.7] w[+mC]=UAS-Cas9.P2}attP40, P{w[+mC]=tubP-GAL80[ts]}10] ; P{w[+mC]=hh-Gal4} / P{y[+t7.7] v[+t1.8]=TRiP.HMS01541}attP2* |
|  | C, right fourth row | hh-Gal4, tubGal80[ts] > Cas9.P2, wg+intergenic sgRNA, UAS:Rbf RNAi | *w ; P{y[+t7.7] w[+mC]=UAS-wg[2x]-intergenic[2x].pCFD6}attP40 / P{y[+t7.7] w[+mC]=UAS-Cas9.P2}attP40, P{w[+mC]=tubP-GAL80[ts]}10] ; P{w[+mC]=hh-Gal4} / P{w[+mC]=UAS-Rbf.D}III* |
|  | C, right bottom | hh-Gal4, tubGal80[ts] > Cas9.P2, wg+intergenic sgRNA, cycA RNAi | *w ; P{y[+t7.7] w[+mC]=UAS-wg[2x]-intergenic[2x].pCFD6}attP40 / P{y[+t7.7] w[+mC]=UAS-Cas9.P2}attP40, P{w[+mC]=tubP-GAL80[ts]}10] ; P{w[+mC]=hh-Gal4} / P{y[+t7.7] v[+t1.8]=TRiP.GLV21059}attP2* |
|  |  |  |  |
|  | D, left top | hh-Gal4, tubGal80[ts] > Cas9.P2, wg+intergenic sgRNA, yki[ACT] | *w ; P{y[+t7.7] w[+mC]=UAS-wg[2x]-intergenic[2x].pCFD6}attP40 / P{y[+t7.7] w[+mC]=UAS-Cas9.P2}attP40, P{w[+mC]=tubP-GAL80[ts]}10] ; P{w[+mC]=hh-Gal4} / P{y[+t7.7] w[+mC]=UAS-yki.S111A.S168A.S250A.V5}attP2* |
|  | D, left second row | hh-Gal4, tubGal80[ts] > Cas9.P2, wg+intergenic sgRNA, UAS:EGFR[gammatop] | *w ; P{y[+t7.7] w[+mC]=UAS-wg[2x]-intergenic[2x].pCFD6}attP40 / P{y[+t7.7] w[+mC]=UAS-Cas9.P2}attP40, P{w[+mC]=tubP-GAL80[ts]}10] ; P{w[+mC]=hh-Gal4} / P{w[+mC]=UAS-Egfr.lambdatop}3* |
|  | D, left third row | hh-Gal4, tubGal80[ts] > Cas9.P2, wg+intergenic sgRNA, UAS:hh[GFP] | *w ; P{y[+t7.7] w[+mC]=UAS-wg[2x]-intergenic[2x].pCFD6}attP40 / P{y[+t7.7] w[+mC]=UAS-Cas9.P2}attP40, P{w[+mC]=tubP-GAL80[ts]}10] ; P{w[+mC]=hh-Gal4} / P{w[+mC]=UAS-hh.EGFP.H}3* |
|  | D, left fourth row | hh-Gal4, tubGal80[ts] > Cas9.P2, wg+intergenic sgRNA, ci RNAi | *w ; P{y[+t7.7] w[+mC]=UAS-wg[2x]-intergenic[2x].pCFD6}attP40 / P{y[+t7.7] w[+mC]=UAS-Cas9.P2}attP40, P{w[+mC]=tubP-GAL80[ts]}10] ; P{w[+mC]=hh-Gal4} / P{y[+t7.7] v[+t1.8]=TRiP.HMC05801}attP2* |
|  | D, left fifth row | hh-Gal4, tubGal80[ts] > Cas9.P2, wg+intergenic sgRNA, UAS:Dpp | *w ; P{y[+t7.7] w[+mC]=UAS-wg[2x]-intergenic[2x].pCFD6}attP40 / P{y[+t7.7] w[+mC]=UAS-Cas9.P2}attP40, P{w[+mC]=tubP-GAL80[ts]}10] ; P{w[+mC]=hh-Gal4} / P{w[+mC]=UAS-dpp.S}42B.4* |
|  | D, left sixth row | hh-Gal4, tubGal80[ts] > Cas9.P2, wg+intergenic sgRNA, UAS:tkv[act] | *w ; P{y[+t7.7] w[+mC]=UAS-wg[2x]-intergenic[2x].pCFD6}attP40 / P{y[+t7.7] w[+mC]=UAS-Cas9.P2}attP40, P{w[+mC]=tubP-GAL80[ts]}10] ; P{w[+mC]=hh-Gal4} / P{w[+mC]=UAS-tkv.Q253D.Nb}3* |
|  | D, left seventh row | hh-Gal4, tubGal80[ts] > Cas9.P2, wg+intergenic sgRNA, UAS:Bsk[DN] | *w ; P{y[+t7.7] w[+mC]=UAS-wg[2x]-intergenic[2x].pCFD6}attP40 / P{y[+t7.7] w[+mC]=UAS-Cas9.P2}attP40, P{w[+mC]=tubP-GAL80[ts]}10] ; P{w[+mC]=hh-Gal4} / P{w[+mC]=UAS-bsk.K53R}20.1a* |
|  | D, right top | hh-Gal4, tubGal80[ts] > Cas9.P2, wg+intergenic sgRNA, UAS:upd | *w ; P9(UAS-upd)26.2 / P{y[+t7.7] w[+mC]=UAS-Cas9.P2}attP40, P{w[+mC]=tubP-GAL80[ts]}10] ; P{w[+mC]=hh-Gal4} / P{y[+t7.7] w[+mC]=UAS-wg[2x]-intergenic[2x].pCFD6}attP2* |
|  | D, right second row | hh-Gal4, tubGal80[ts] > Cas9.P2, wg+intergenic sgRNA, stat92E RNAi | *w ; P{y[+t7.7] w[+mC]=UAS-wg[2x]-intergenic[2x].pCFD6}attP40 / P{y[+t7.7] w[+mC]=UAS-Cas9.P2}attP40, P{w[+mC]=tubP-GAL80[ts]}10] ; P{w[+mC]=hh-Gal4} / P{y[+t7.7] v[+t1.8]=TRiP.HMS00035}attP2* |
|  | D, right third row | hh-Gal4, tubGal80[ts] > Cas9.P2, wg+intergenic sgRNA, UAS:Notch[ACT] | *w ; P{y[+t7.7] w[+mC]=UAS-wg[2x]-intergenic[2x].pCFD6}attP40 / P{y[+t7.7] w[+mC]=UAS-Cas9.P2}attP40, P{w[+mC]=tubP-GAL80[ts]}10] ; P{w[+mC]=hh-Gal4} / P{w[+mC]=UAS-Delta::N.DeltaECN}B2a3* |
|  | D, right fourth row | hh-Gal4, tubGal80[ts] > Cas9.P2, wg+intergenic sgRNA, Notch RNAi | *w ; P{y[+t7.7] w[+mC]=UAS-wg[2x]-intergenic[2x].pCFD6}attP40 / P{y[+t7.7] w[+mC]=UAS-Cas9.P2}attP40, P{w[+mC]=tubP-GAL80[ts]}10] ; P{w[+mC]=hh-Gal4} / P{y[+t7.7] v[+t1.8]=TRiP.HMS00001}attP2* |
|  | D, right fifth row | hh-Gal4, tubGal80[ts] > Cas9.P2, wg+intergenic sgRNA, Myc[ACT] | *w ; P{y[+t7.7] w[+mC]=UAS-wg[2x]-intergenic[2x].pCFD6}attP40 / P{y[+t7.7] w[+mC]=UAS-Cas9.P2}attP40, P{w[+mC]=tubP-GAL80[ts]}10] ; P{w[+mC]=hh-Gal4} / M{w[+mC]=UAS-Myc.HA.WT}ZH-86Fb* |
|  | D, right sixth row | hh-Gal4, tubGal80[ts] > Cas9.P2, wg+intergenic sgRNA, Myc RNAi | *w ; P{y[+t7.7] w[+mC]=UAS-wg[2x]-intergenic[2x].pCFD6}attP40 / P{y[+t7.7] w[+mC]=UAS-Cas9.P2}attP40, P{w[+mC]=tubP-GAL80[ts]}10] ; P{w[+mC]=hh-Gal4} / P{y[+t7.7] v[+t1.8]=TRiP.HMS01538}attP2* |
|  |  |  |  |
| Figure S7 | A, left | hh-Gal4, ex-LacZ > mCherry RNAi | *w ; P{w[+mC]=lacW}ex[697] / P{w[+mC]=hh-Gal4} / P{y[+t7.7] v[+t1.8]=VALIUM20-mCherry.RNAi}attP2* |
|  | A, right | hh-Gal4, ex-LacZ > wg RNAi | *w ; P{w[+mC]=lacW}ex[697] / P{w[+mC]=hh-Gal4} / P{y[+t7.7] v[+t1.8]=TRiP.HMS00794}attP2* |
|  |  |  |  |
|  | B, left | hh-Gal4, puc:lacZ > UAS:Cas9.P2, no guide | *w ;; P{w[+mC]=hh-Gal4} / puc-LacZ* |
|  | B, right | hh-Gal4, puc:lacZ > UAS:Cas9.P2, wg+wnt6 sgRNA | *w ; P{y[+t7.7] w[+mC]=UAS-wg[2x]-wnt6[2x].pCFD6}attP40 / P{w[+mC]=hh-Gal4} / puc-LacZ* |
|  |  |  |  |
|  | C, left | hh-Gal4, tubGal80[ts] > Cas9.P2, wg+intergenic sgRNA | *w ; P{y[+t7.7] w[+mC]=UAS-wg[2x]-intergenic[2x].pCFD6}attP40 / P{y[+t7.7] w[+mC]=UAS-Cas9.P2}attP40, P{w[+mC]=tubP-GAL80[ts]}10] ; P{w[+mC]=hh-Gal4}* |
|  | C, right | hh-Gal4, tubGal80[ts] > Cas9.P2, wg+intergenic sgRNA | *w ; P{y[+t7.7] w[+mC]=UAS-wg[2x]-intergenic[2x].pCFD6}attP40 / P{y[+t7.7] w[+mC]=UAS-Cas9.P2}attP40, P{w[+mC]=tubP-GAL80[ts]}10] ; P{w[+mC]=hh-Gal4} / P{w[+mC]=UAS-puc.M}3* |
|  |  |  |  |
| Figure S8 | left, top | hh-Gal4, tubGal80[ts] > Cas9.P2, wg+intergenic sgRNA | *w ; P{y[+t7.7] w[+mC]=UAS-wg[2x]-intergenic[2x].pCFD6}attP40 / P{y[+t7.7] w[+mC]=UAS-Cas9.P2}attP40, P{w[+mC]=tubP-GAL80[ts]}10] ; P{w[+mC]=hh-Gal4}* |
|  | left, second row | hh-Gal4, tubGal80[ts] > Cas9.P2, wg+intergenic sgRNA, hid RNAi GD8269 | *w ; P{y[+t7.7] w[+mC]=UAS-wg[2x]-intergenic[2x].pCFD6}attP40 / P{y[+t7.7] w[+mC]=UAS-Cas9.P2}attP40, P{w[+mC]=tubP-GAL80[ts]}10] ; P{w[+mC]=hh-Gal4} / P{GD8269}* |
|  | left, third row | hh-Gal4, tubGal80[ts] > Cas9.P2, wg+intergenic sgRNA, hid RNAi GD7912 | *w ; P{y[+t7.7] w[+mC]=UAS-wg[2x]-intergenic[2x].pCFD6}attP40 / P{y[+t7.7] w[+mC]=UAS-Cas9.P2}attP40, P{w[+mC]=tubP-GAL80[ts]}10] ; P{w[+mC]=hh-Gal4} / P{GD7912}* |
|  | left, bottom | hh-Gal4, tubGal80[ts] > Cas9.P2, wg+intergenic sgRNA, grim RNAi | *w ; P{GD21830} / P{y[+t7.7] w[+mC]=UAS-Cas9.P2}attP40, P{w[+mC]=tubP-GAL80[ts]}10] ; P{w[+mC]=hh-Gal4} / P{y[+t7.7] w[+mC]=UAS-wg[2x]-intergenic[2x].pCFD6}attP2* |
|  | right, top | hh-Gal4, tubGal80[ts] > Cas9.P2, wg+intergenic sgRNA, rpr RNAi GD12050 | *w ; P{y[+t7.7] w[+mC]=UAS-wg[2x]-intergenic[2x].pCFD6}attP40 / P{y[+t7.7] w[+mC]=UAS-Cas9.P2}attP40, P{w[+mC]=tubP-GAL80[ts]}10] ; P{w[+mC]=hh-Gal4} / P{GD12050}* |
|  | right, second row | hh-Gal4, tubGal80[ts] > Cas9.P2, wg+intergenic sgRNA, rpr RNAi KK101234 | *w ; P{KK101234}30B / P{y[+t7.7] w[+mC]=UAS-Cas9.P2}attP40, P{w[+mC]=tubP-GAL80[ts]}10] ; P{w[+mC]=hh-Gal4} / P{y[+t7.7] w[+mC]=UAS-wg[2x]-intergenic[2x].pCFD6}attP2* |
|  | right, third row | hh-Gal4, tubGal80[ts] > Cas9.P2, wg+intergenic sgRNA, rpr RNAi BL51846 | *w ; P{y[+t7.7] v[+t1.8]=TRiP.HMC03419}attP40 / P{y[+t7.7] w[+mC]=UAS-Cas9.P2}attP40, P{w[+mC]=tubP-GAL80[ts]}10] ; P{w[+mC]=hh-Gal4} / P{y[+t7.7] w[+mC]=UAS-wg[2x]-intergenic[2x].pCFD6}attP2* |
|  | right, fourth row | hh-Gal4, tubGal80[ts] > Cas9.P2, wg+intergenic sgRNA, skl RNAi GD7172 | *w ; P{y[+t7.7] w[+mC]=UAS-wg[2x]-intergenic[2x].pCFD6}attP40 / P{y[+t7.7] w[+mC]=UAS-Cas9.P2}attP40, P{w[+mC]=tubP-GAL80[ts]}10] ; P{w[+mC]=hh-Gal4} / P{GD7172}* |
|  | right, fifth row | hh-Gal4, tubGal80[ts] > Cas9.P2, wg+intergenic sgRNA, skl RNAi BL102512 | *w ; P{y[+t7.7] w[+mC]=UAS-wg[2x]-intergenic[2x].pCFD6}attP40 / P{y[+t7.7] w[+mC]=UAS-Cas9.P2}attP40, P{w[+mC]=tubP-GAL80[ts]}10] ; P{w[+mC]=hh-Gal4} / P{KK102512}* |
|  | right, sixth row | hh-Gal4, tubGal80[ts] > Cas9.P2, wg+intergenic sgRNA, skl RNAi BL28678 | *w ; P{y[+t7.7] w[+mC]=UAS-wg[2x]-intergenic[2x].pCFD6}attP40 / P{y[+t7.7] w[+mC]=UAS-Cas9.P2}attP40, P{w[+mC]=tubP-GAL80[ts]}10] ; P{w[+mC]=hh-Gal4} / P{y[+t7.7] v[+t1.8]=TRiP.JF03093}attP2* |
|  |  |  |  |
| Figure S9 | left | hh-Gal4, tubGal80[ts] > w | *w ; P{w[+mC]=tubP-GAL80[ts]}10] ; P{w[+mC]=hh-Gal4}* |
|  | second from left | hh-Gal4, tubGal80[ts] > wg RNAi | *w / y[1] sc[*] v[1] sev[21] ; P{w[+mC]=tubP-GAL80[ts]}10]; P{y[+t7.7] v[+t1.8]=TRiP.HMS00794}attP2 / P{w[+mC]=hh-Gal4}* |
|  | third from left | hh-Gal4, tubGal80[ts] > arm RNAi | *w / y[1] sc[*] v[1] sev[21] ; P{w[+mC]=tubP-GAL80[ts]}10]; P{y[+t7.7] v[+t1.8]=TRiP.HMS01414}attP2 / P{w[+mC]=hh-Gal4}* |
|  | right | hh-Gal4, tubGal80[ts] > UAS:wg | *w ; P{w[+mC]=tubP-GAL80[ts]}10]; P{w[+mC]=UAS-wg.H.T:HA1}6C / P{w[+mC]=hh-Gal4}* |
|  |  |  |  |
| Figure S10 | top row | hh-Gal4, tubGal80[ts] > UAS:p53 | *w ; P{w[+mC]=tubP-GAL80[ts]}10] / UAS:p53 ; P{w[+mC]=hh-Gal4}* |
|  | middle row | hh-Gal4, tubGal80[ts] > UAS:wg | *w ; P{w[+mC]=tubP-GAL80[ts]}10] ; P{w[+mC]=hh-Gal4} / P{w[+mC]=UAS-wg.H.T:HA1}6C* |
|  | bottom row | hh-Gal4, tubGal80[ts] > UAS:p53 + UAS:wg | *w ; P{w[+mC]=tubP-GAL80[ts]}10] / UAS:p53 ; P{w[+mC]=hh-Gal4} / P{w[+mC]=UAS-wg.H.T:HA1}6C* |
