# Supplementary material for: Wnt signaling modulates the response to DNA damage in the Drosophila wing imaginal disc by regulating the EGFR pathway
Source: PLoS Biol. 2024 Jul 24;22(7):e3002547. doi: 10.1371/journal.pbio.3002547 (PMC11341097; doi:10.1371/journal.pbio.3002547)
Supplement: S1 Fig — (A) Somatic single CRISPR KOs of each Wnt ligand in the posterior of the developing wing. Single KO of wg produces a loss of the wing margin in the posterior, whereas no other Wnt ligand displays a phenotype. (B) Double CRISPR KOs of each pairwise comparison of Wnt ligands using hh-Gal4. In combination with any other Wnt ligand, wg causes a dramatic defect in wing development, indicative of excessive cell death. All other pairwise combinations appear wild type. (C) Double CRISPR KO of wg with 2 separate intergenic sgRNA sequences causes severe wing defects, whereas double KO of an intergenic sequence with wnt2 or wnt10 produces no phenotype. The phenotype of wntless single KO is reminiscent of wg KO alone. (D) Scoring of wing defects shown in (A-C). Posterior is down in all wing images. The data underlying the graphs shown in the figure can be found in S1 Data. (DOCX) [file pbio.3002547.s004.docx]

**
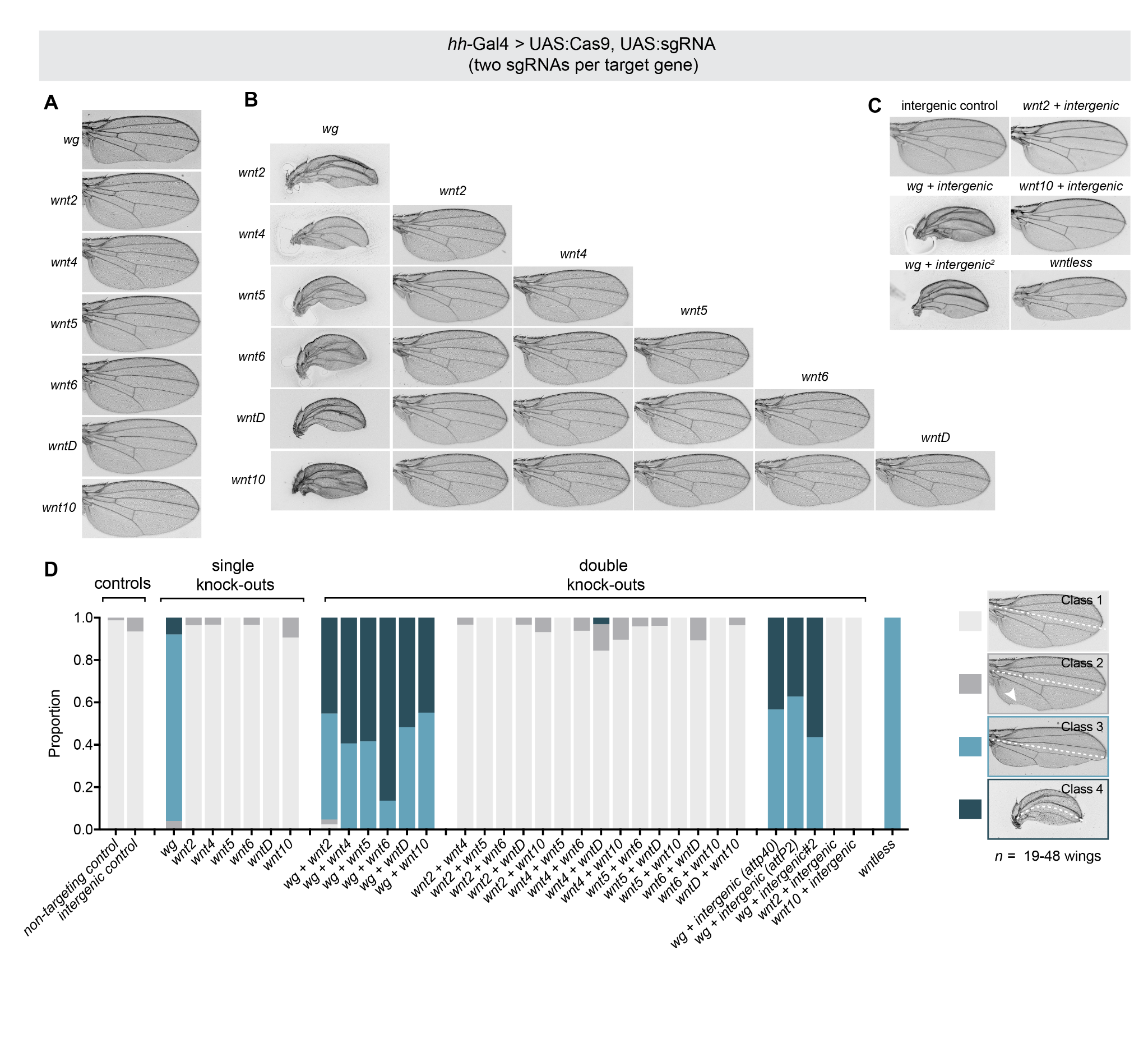
**

**Figure S1 (related to Figure 1). CRISPR knock-out of *wg* sensitizes developing wing tissue to DNA damage.** (A) Somatic single CRISPR KOs of each Wnt ligand in the posterior of the developing wing. Single KO of *wg* produces a loss of the wing margin in the posterior, whereas no other Wnt ligand displays a phenotype. (B) Double CRISPR KOs of each pairwise comparison of Wnt ligands using *hh-Gal4*. In combination with any other Wnt ligand, *wg* causes a dramatic defect in wing development, indicative of excessive cell death. All other pairwise combinations appear wildtype. (C) Double CRISPR KO of *wg* with two separate intergenic sgRNA sequences causes severe wing defects, whereas double KO of an intergenic sequence with *wnt2* or *wnt10* produces no phenotype. The phenotype of *wntless* single KO is reminiscent of *wg* KO alone. (D) Scoring of wing defects shown in A-C. Posterior is down in all wing images.
