# Supplementary material for: Wnt signaling modulates the response to DNA damage in the Drosophila wing imaginal disc by regulating the EGFR pathway
Source: PLoS Biol. 2024 Jul 24;22(7):e3002547. doi: 10.1371/journal.pbio.3002547 (PMC11341097; doi:10.1371/journal.pbio.3002547)
Supplement: S2 Fig — Wing discs of the indicated genotypes stained for Dcp1 to visualize apoptotic cells. The adult phenotypes shown in S1 Fig correspond to increased apoptosis in the posterior of the wing discs. (DOCX) [file pbio.3002547.s005.docx]

**
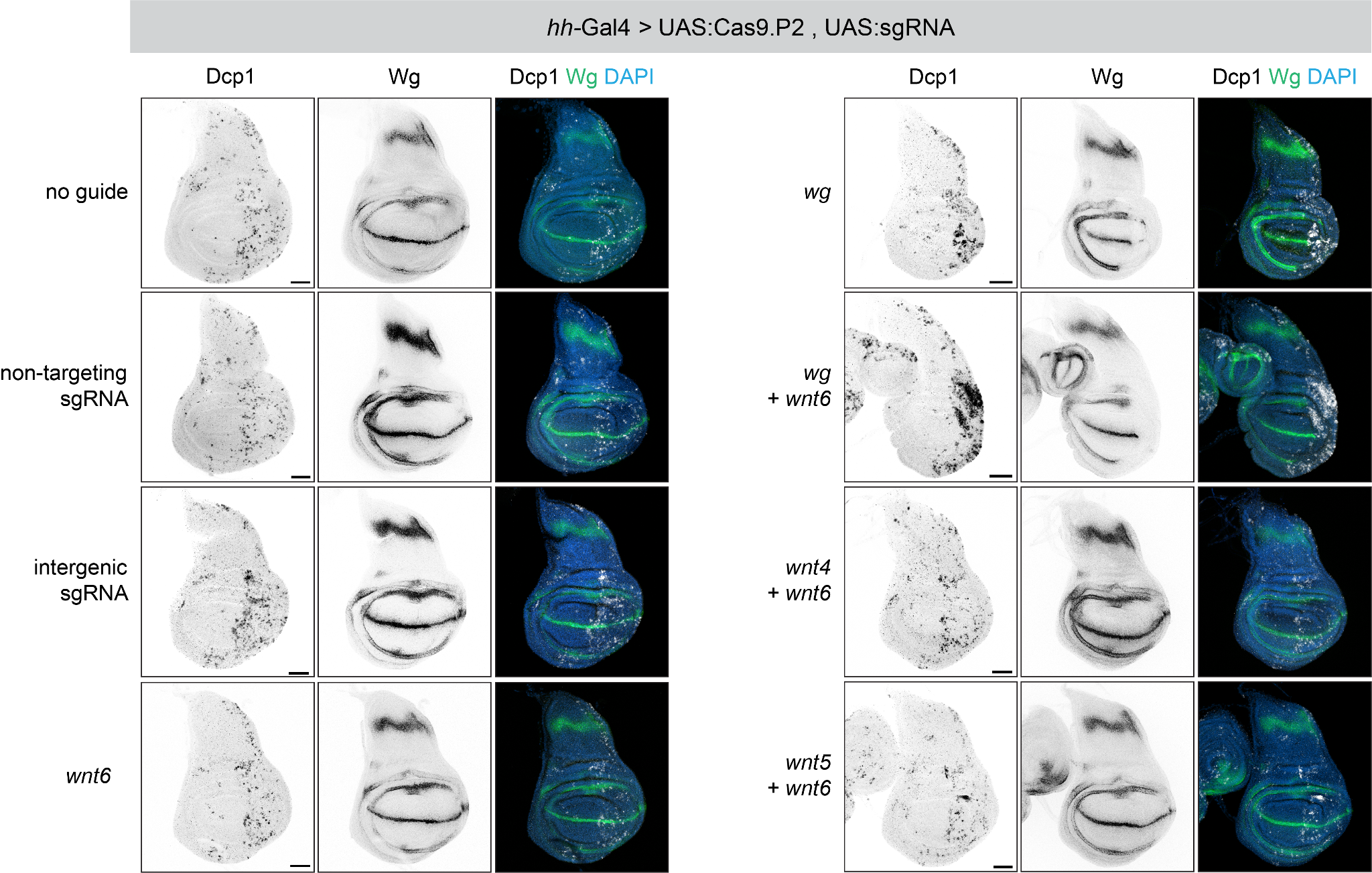
**

**Figure S2. (Related to Figure 1) Apoptosis in CRISPR KO wing discs.** Wing discs of the indicated genotypes stained for Dcp1 to visualize apoptotic cells. The adult phenotypes shown in Figure S1 correspond to increased apoptosis in the posterior of the wing discs. Posterior is to the right, dorsal is up. Scale bar = 50 µm.
