# Supplementary material for: Wnt signaling modulates the response to DNA damage in the Drosophila wing imaginal disc by regulating the EGFR pathway
Source: PLoS Biol. 2024 Jul 24;22(7):e3002547. doi: 10.1371/journal.pbio.3002547 (PMC11341097; doi:10.1371/journal.pbio.3002547)
Supplement: S3 Fig — (A) As an alternative to hh-Gal4, nub-Gal4 driving UAS:Cas9.P2 throughout the wing pouch sensitizes cells to DNA damage caused by CRISPR targeting of an intergenic region. (B) wg RNAi in the disc posterior sensitizes wing disc cells to DNA damage caused by CRISPR targeting of an intergenic region, and (C) against a sgRNA targeting the yellow gene, which has no apoptotic phenotype by itself. (D) hh-Gal4 driving a lower-toxicity variant of Cas9, uMCas9, also sensitizes wing disc cells to DNA damage caused by CRISPR. (E) Somatic CRISPR in the wing disc using a lower-toxicity variant of Cas9, uMCas9, causes substantial apoptosis with a wide variety of sgRNAs targeting intergenic sequences, genes expressed in the wing disc, and genes not expressed in the wing disc (osk). P values are shown from Student t test, with Welch correction for any comparison with unequal variances. The data underlying the graphs shown in the figure can be found in S1 Data. (DOCX) [file pbio.3002547.s006.docx]

**
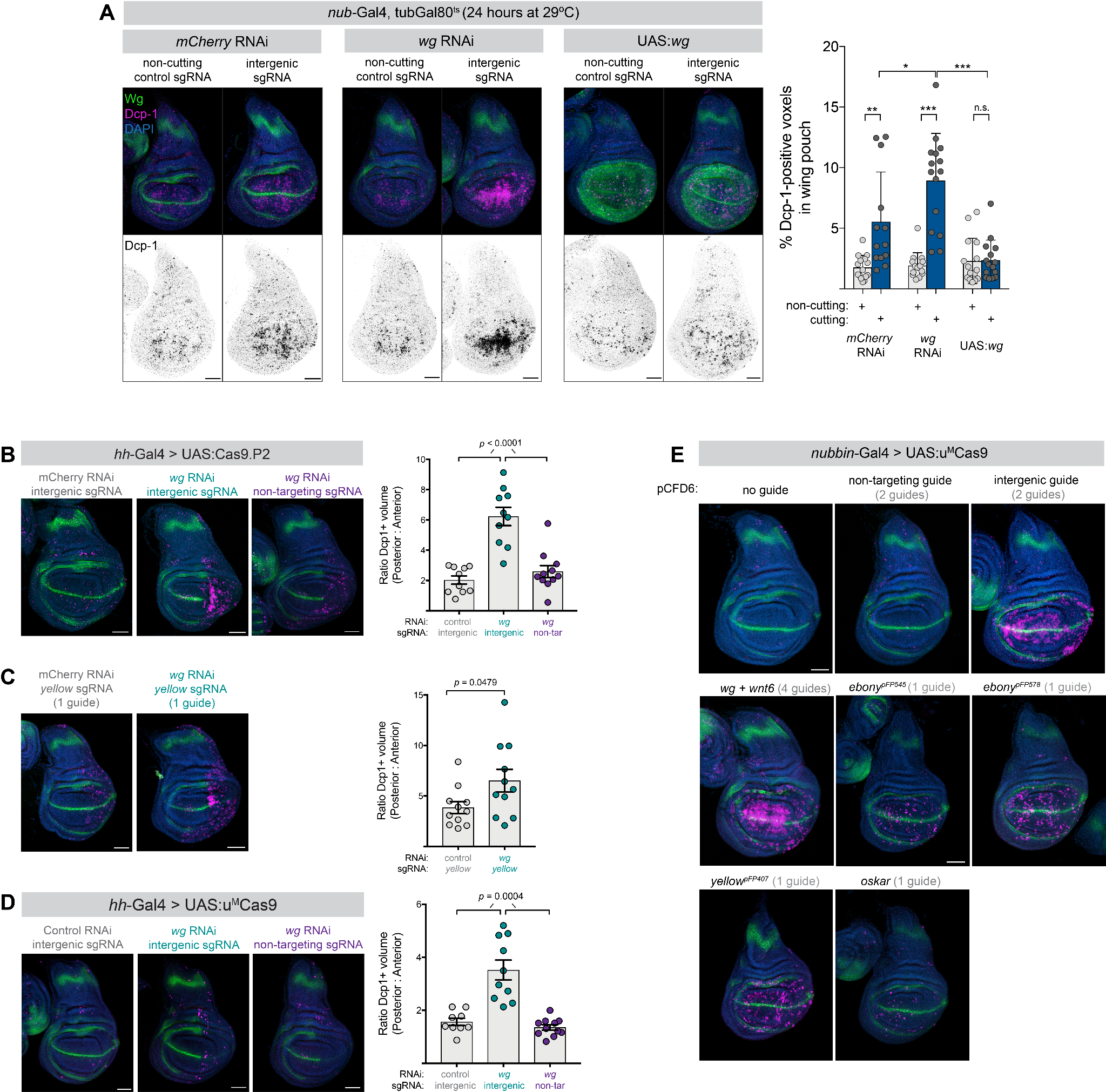
**

**Figure S3. (Related to Figure 1). Additional validation that *wg* signaling modulates the response to DNA damage caused by somatic CRISPR in the wing disc.** (A) As an alternative to *hh-Gal4, nub-Gal4* driving UAS:Cas9.P2 throughout the wing pouch sensitizes cells to DNA damage caused by CRISPR targeting of an intergenic region. (B) *wg* RNAi in the disc posterior sensitizes wing disc cells to DNA damage caused by CRISPR targeting of an intergenic region, and (C) against a sgRNA targeting the *yellow* gene which has no apoptotic phenotype by itself. (D) *hh-Gal4* driving a lower-toxicity variant of Cas9, u^M^Cas9, also sensitizes wing disc cells to DNA damage caused by CRISPR. (E) Somatic CRISPR in the wing disc using a lower-toxicity variant of Cas9, u^M^Cas9, causes substantial apoptosis with a wide variety of sgRNAs targeting intergenic sequences, genes expressed in the wing disc, and genes not expressed in the wing disc (*osk*). *p-*values are shown from student’s t-test, with Welch’s correction for any comparison with unequal variances. Posterior is to the right, dorsal is up. Scale bar = 50 µm.
