# Supplementary material for: Wnt signaling modulates the response to DNA damage in the Drosophila wing imaginal disc by regulating the EGFR pathway
Source: PLoS Biol. 2024 Jul 24;22(7):e3002547. doi: 10.1371/journal.pbio.3002547 (PMC11341097; doi:10.1371/journal.pbio.3002547)
Supplement: S4 Fig — hh-Gal4 or hh-Gal4, tubGal80ts was used to drive RNAi against wg or arm, respectively, and larvae were subjected to 1,000 RADs of X-rays 4 hours prior to dissection. The amount of apoptosis was quantified by measuring the percentage of Dcp1+ voxels in the posterior (Gal4 on) versus anterior (Gal4 off). In the case of wg RNAi, the effect of wg RNAi on apoptosis levels appeared more pronounced in the notum rather than the wing pouch. The data underlying the graphs shown in the figure can be found in S1 Data. (DOCX) [file pbio.3002547.s007.docx]

**
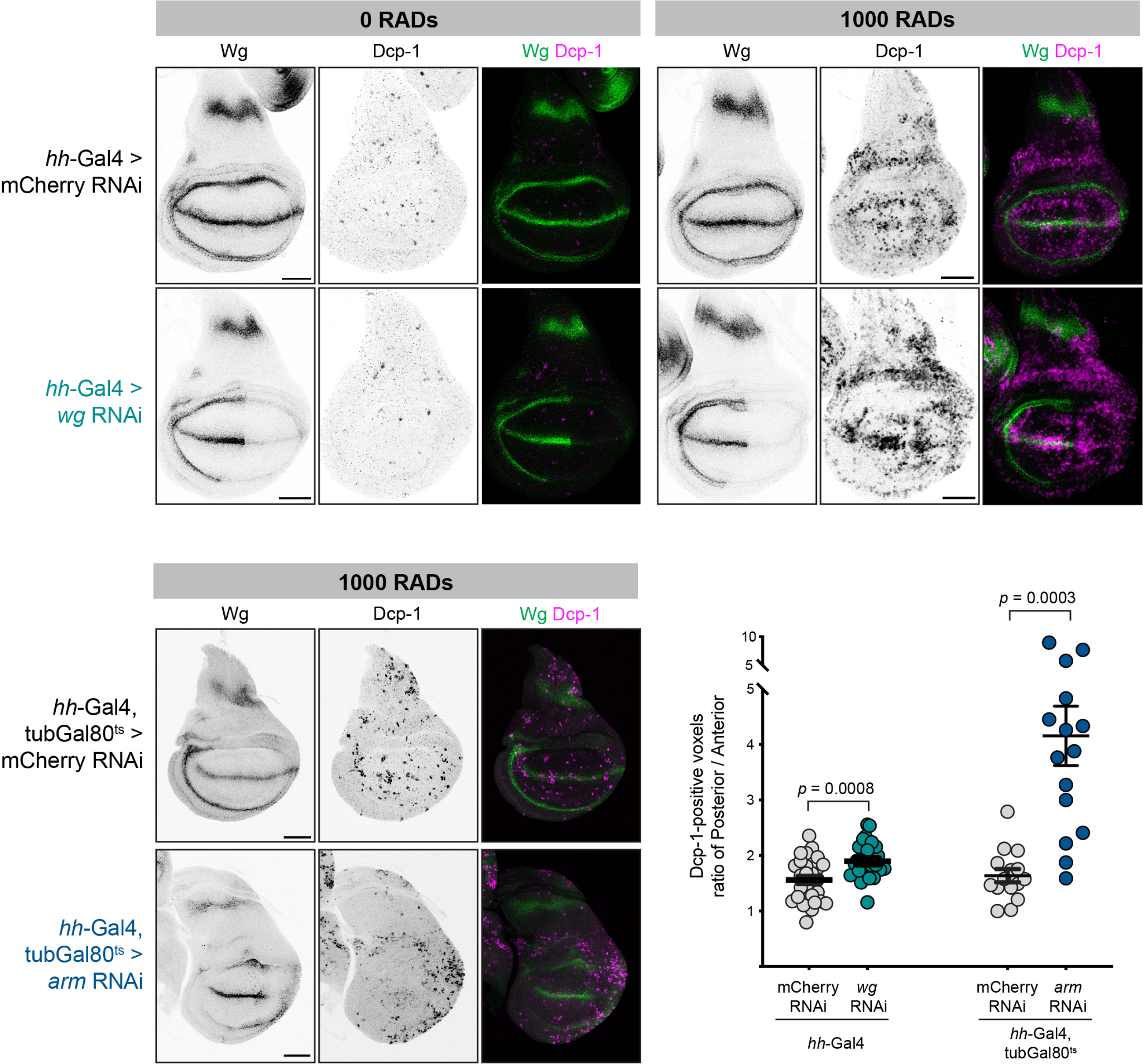
**

**Figure S4. (Related to Figure 2.) RNAi against *wg* or *arm* sensitizes wing discs to 1000 RADs of X-ray damage.** *hh-Gal4* or *hh-Gal4*, *tubGal80^ts^* was used to drive RNAi against *wg* or *arm* respectively, and larvae were subjected to 1000 RADs of X-rays four hours prior to dissection. The amount of apoptosis was quantified by measuring the percentage of Dcp1+ voxels in the posterior (Gal4 on) versus anterior (Gal4 off). In the case of *wg* RNAi, the effect of *wg* RNAi on apoptosis levels appeared more pronounced in the notum rather than the wing pouch.
