# Supplementary material for: Wnt signaling modulates the response to DNA damage in the Drosophila wing imaginal disc by regulating the EGFR pathway
Source: PLoS Biol. 2024 Jul 24;22(7):e3002547. doi: 10.1371/journal.pbio.3002547 (PMC11341097; doi:10.1371/journal.pbio.3002547)
Supplement: S5 Fig — wg was overexpressed in the posterior wing disc using en-Gal4, tubGal80ts > UAS:dCas9-VPR, and flies were subjected to DNA damage caused by (A) 1,000 RADs of X-rays 4 hours prior to dissection, (B) pirarubicin for 24 hours, or (C) cisplatin for 24 hours. Dotted lines represent the approximate boundary of the posterior compartment in control discs (identified via UAS:GFP expression) or the regions where excess Wg is detected via antibody staining in CRISPRa tissues. Scale bars are 50 μm, posterior is the right, and dorsal is up. Wg signal is displayed using the “Fire” lookup table in FIJI/ImageJ. P values are shown from Student t test, with Welch correction for any comparison with unequal variances. The data underlying the graphs shown in the figure can be found in S1 Data. (DOCX) [file pbio.3002547.s008.docx]

**
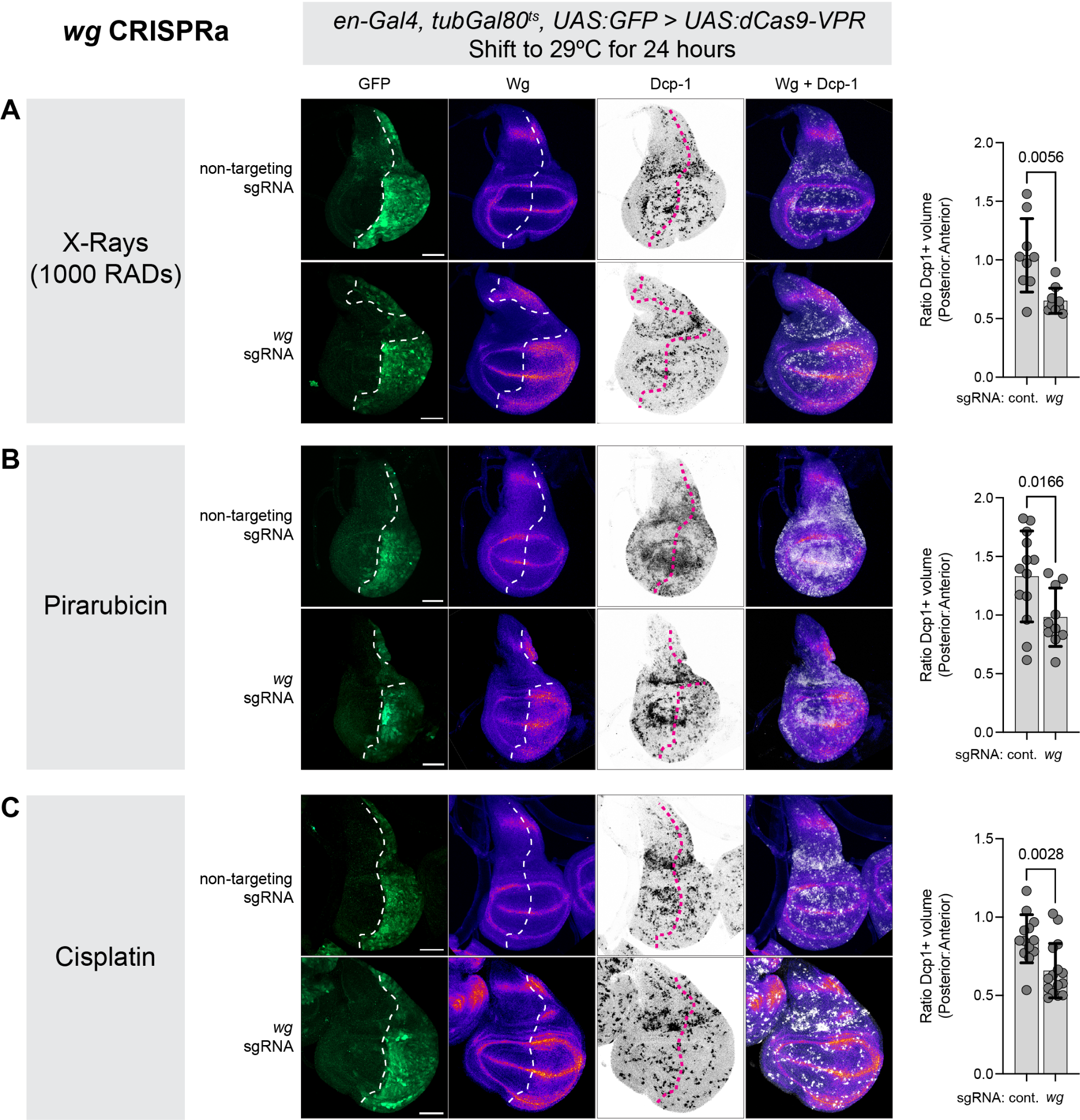
**

**Figure S5. (Related to Figure 2) *wg* overexpression using CRISPRa dampens apoptosis caused by X-rays and DNA-damaging drugs.** *wg* was overexpressed in the posterior wing disc using *en-Gal4, tubGal80^ts^ > UAS:dCas9-VPR* and flies were subjected to DNA damage caused by (A) 1000 RADs of X-rays four hours prior to dissection, (B) pirarubicin for 24 hours, or (C) cisplatin for 24 hours. Dotted lines represent the approximate boundary of the posterior compartment in control discs (identified via UAS:GFP expression) or the regions where excess Wg is detected via antibody staining in CRISPRa tissues. Scale bars are 50µm, posterior is the right, and dorsal is up. Wg signal is displayed using the “Fire” lookup table in FIJI/ImageJ. *p-*values are shown from student’s t-test, with Welch’s correction for any comparison with unequal variances.
