# Supplementary material for: Wnt signaling modulates the response to DNA damage in the Drosophila wing imaginal disc by regulating the EGFR pathway
Source: PLoS Biol. 2024 Jul 24;22(7):e3002547. doi: 10.1371/journal.pbio.3002547 (PMC11341097; doi:10.1371/journal.pbio.3002547)
Supplement: S6 Fig — (A) Schematic of the candidate suppressor screen. hh-Gal4, tubGal80ts > UAS:Cas9.P2 + pCFD6-wg-intergenic was used to drive DNA damage and apoptosis in the posterior wing disc, in the presence of various UAS-driven RNAi or other functional transgenes targeting the DNA damage repair pathway and various signaling pathways. Discs were stained for Dcp1 and a primary screen qualitatively identified major changes in the amount of apoptosis in the wing disc. (B) Control discs show the levels of Dcp1 signal seen in representative discs with a nontargeting sgRNA (negative control) and with sgRNAs targeting wg and an intergenic region (positive control.) Primary screen hits are shown in pink for members of the (C) DNA damage pathway and (D) various signaling pathways. These hits were secondarily screened and quantified as shown in Fig 3. (DOCX) [file pbio.3002547.s009.docx]

**
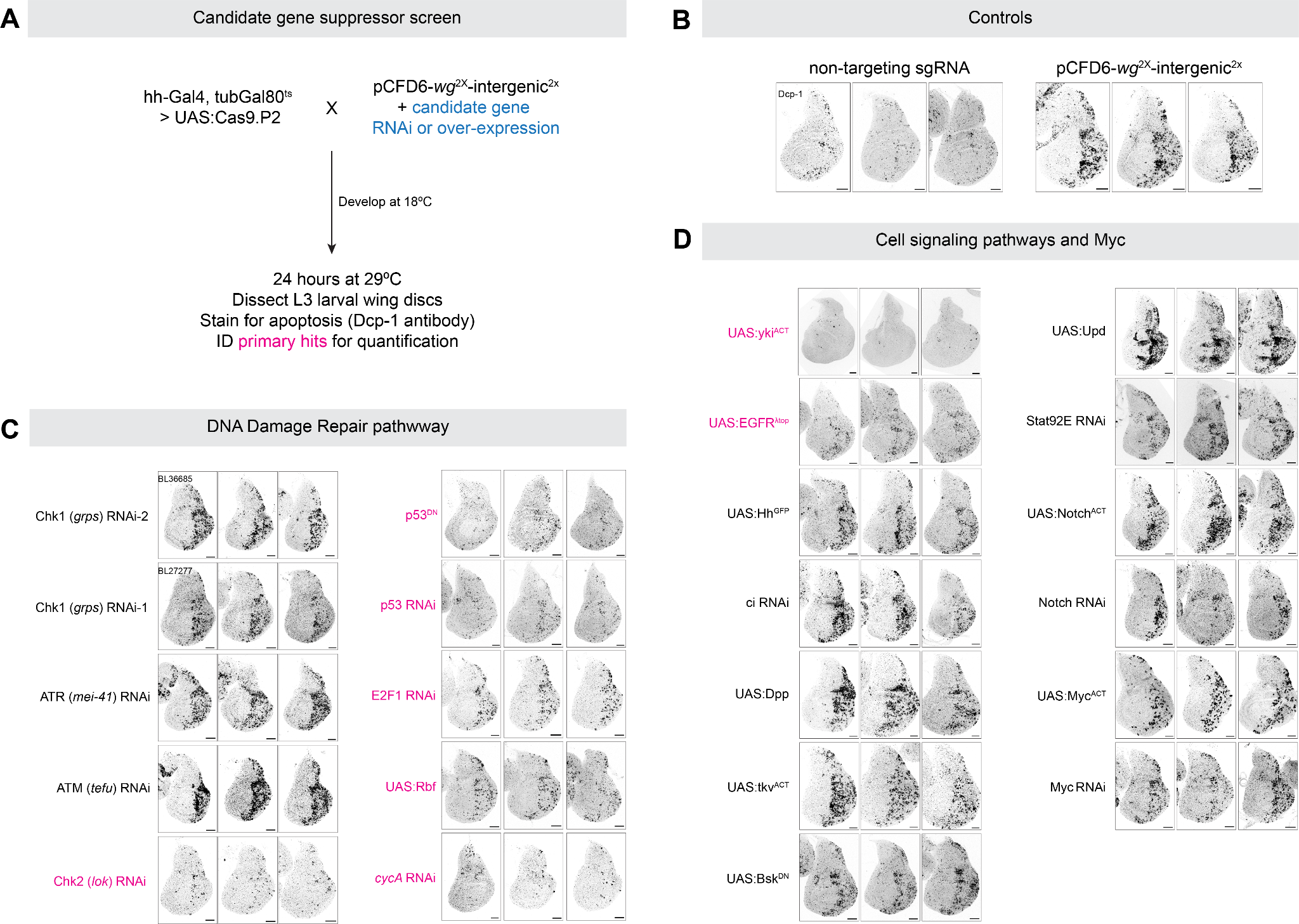
**

**Figure S6. (Related to Figure 3.) Candidate suppressor screen identifies members of the DNA damage response pathway, the Hippo pathway, and the EGFR pathway as suppressors of DNA damage-induced apoptosis in the presence of compromised Wnt signaling.** (A) Schematic of the candidate suppressor screen. *hh-Gal4, tubGal80^ts^ > UAS:Cas9.P2* + pCFD6-*wg-intergenic* was used to drive DNA damage and apoptosis in the posterior wing disc, in the presence of various UAS-driven RNAi or other functional transgenes targeting the DNA damage repair pathway and various signaling pathways. Discs were stained for Dcp1 and a primary screen qualitatively identified major changes in the amount of apoptosis in the wing disc. (B) Control discs show the levels of Dcp1 signal seen in representative discs with a non-targeting sgRNA (negative control) and with sgRNAs targeting *wg* and an intergenic region (positive control.) Primary screen hits are shown in pink for members of the (C) DNA damage pathway and (D) various signaling pathways. These hits were secondarily screened and quantified as shown in Figure 3. Scale bars are 50µm, posterior is the right, and dorsal is up.
