# Supplementary material for: Wnt signaling modulates the response to DNA damage in the Drosophila wing imaginal disc by regulating the EGFR pathway
Source: PLoS Biol. 2024 Jul 24;22(7):e3002547. doi: 10.1371/journal.pbio.3002547 (PMC11341097; doi:10.1371/journal.pbio.3002547)
Supplement: S7 Fig — The same screening format as in S6 Fig was used to screen RNAi lines targeting the DIAP1 inhibits rpr, hid, skl, and grm. Of these constructs, 2 hid RNAi lines suppressed apoptosis in this context. (DOCX) [file pbio.3002547.s010.docx]

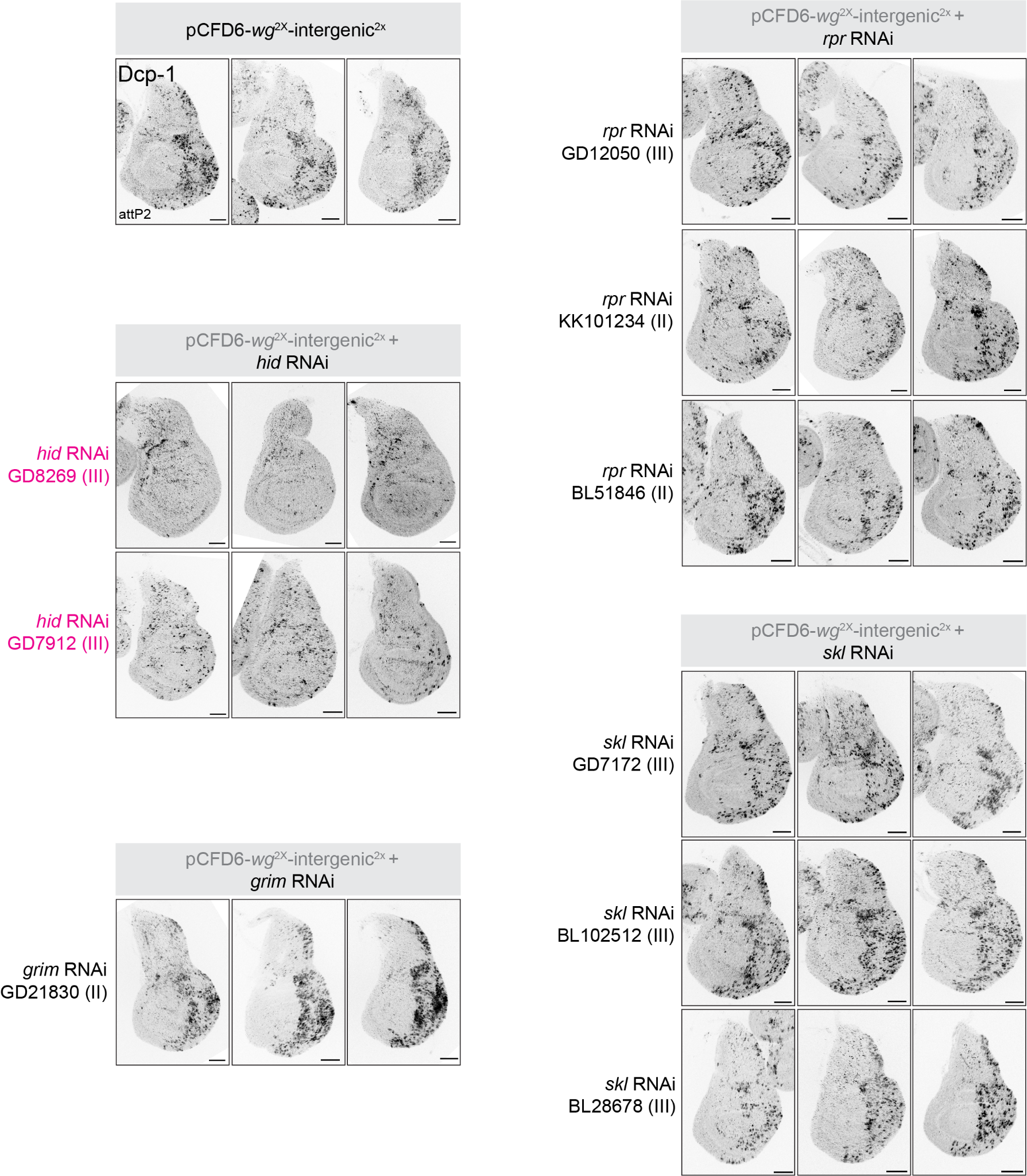


**Figure S7**. (Related to Figure 4.) Candidate suppressor screen identifies hid as the effector of apoptosis in the context of DNA damage in Wnt-compromised discs. The same screening format as in Figure S6 was used to screen RNAi lines targeting the DIAP1 inhibits *rpr*, *hid*, *skl*, and *grm*. Of these constructs, two hid RNAi lines suppressed apoptosis in this context. Scale bars are 50µm, posterior is the right, and dorsal is up.
