# Supplementary material for: Wnt signaling modulates the response to DNA damage in the Drosophila wing imaginal disc by regulating the EGFR pathway
Source: PLoS Biol. 2024 Jul 24;22(7):e3002547. doi: 10.1371/journal.pbio.3002547 (PMC11341097; doi:10.1371/journal.pbio.3002547)
Supplement: S8 Fig — (A) wg RNAi in the posterior wing compartment does not alter the expression of the Hippo signaling reporter ex-LacZ. (B) The JNK signaling reporter puc:lacZ is activated by DNA damage and apoptosis in the wing disc. However, as indicated in S6 Fig, suppressing JNK signaling does not suppress the apoptosis caused by DNA damage in a Wnt-compromised disc. The data underlying the graphs shown in the figure can be found in S1 Data. (DOCX) [file pbio.3002547.s011.docx]

**
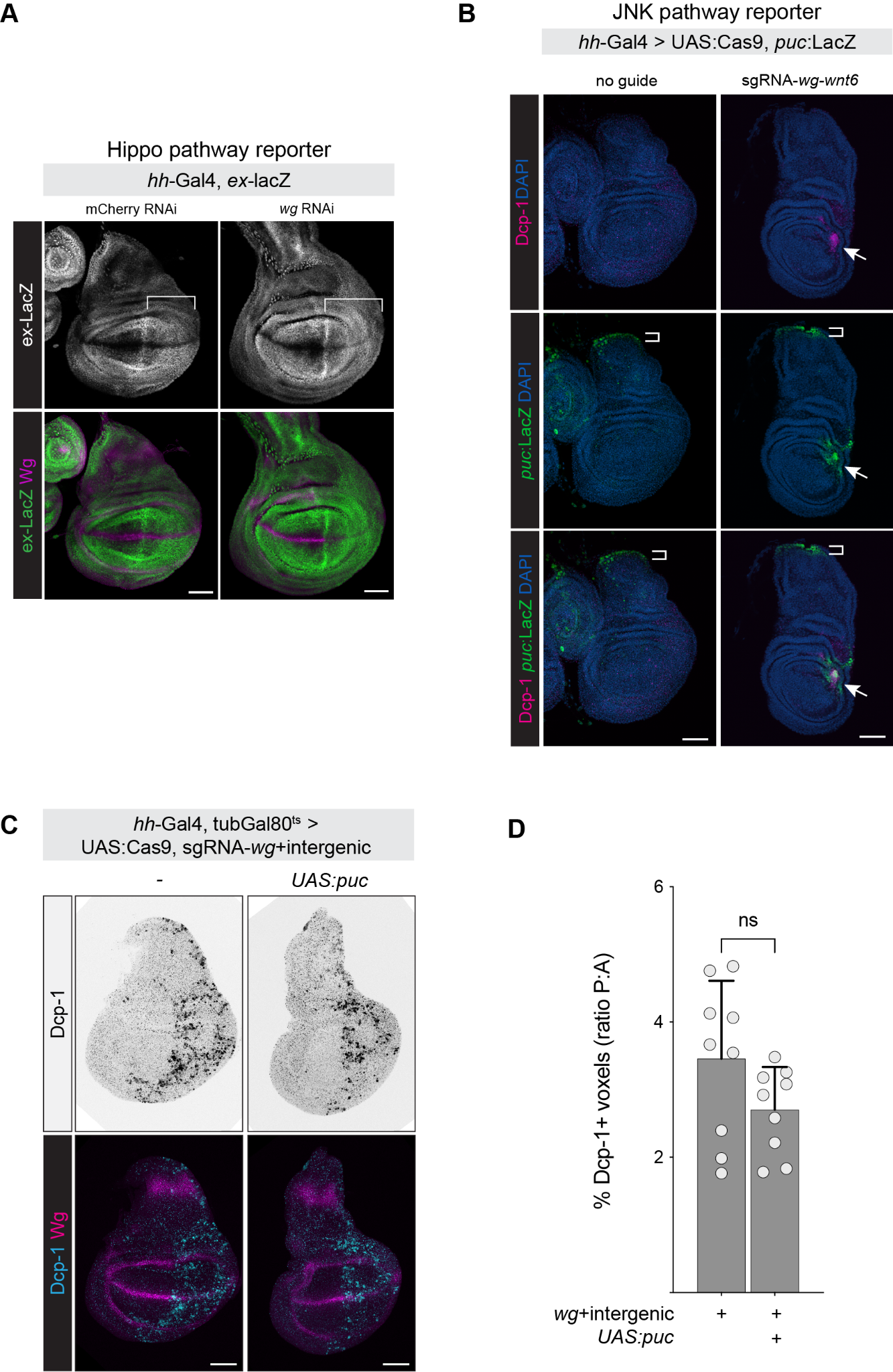
**

**Figure S8. (Related to Figure 3.) The effects of Wnt signaling on the DNA damage response pathway are not mediated via Hippo or JNK signaling.** (A) *wg* RNAi in the posterior wing compartment does not alter the expression of the Hippo signaling reporter *ex-LacZ*. (B) The JNK signaling reporter *puc:lacZ* is activated by DNA damage and apoptosis in the wing disc. However, as indicated in Figure S6, suppressing JNK signaling does not suppress the apoptosis caused by DNA damage in a Wnt-compromised disc. (C) Over-expression of the *puc*, a negative regulator of JNK signaling, does not reduce apoptosis caused by DNA damage in a Wnt-compromised disc. (D) Quantification of the experiment shown in C. Scale bars are 50µm, posterior is the right, and dorsal is up.
