# Supplementary material for: Wnt signaling modulates the response to DNA damage in the Drosophila wing imaginal disc by regulating the EGFR pathway
Source: PLoS Biol. 2024 Jul 24;22(7):e3002547. doi: 10.1371/journal.pbio.3002547 (PMC11341097; doi:10.1371/journal.pbio.3002547)
Supplement: S9 Fig — In situ hybridization against vn in the indicated genotypes. (DOCX) [file pbio.3002547.s012.docx]

**
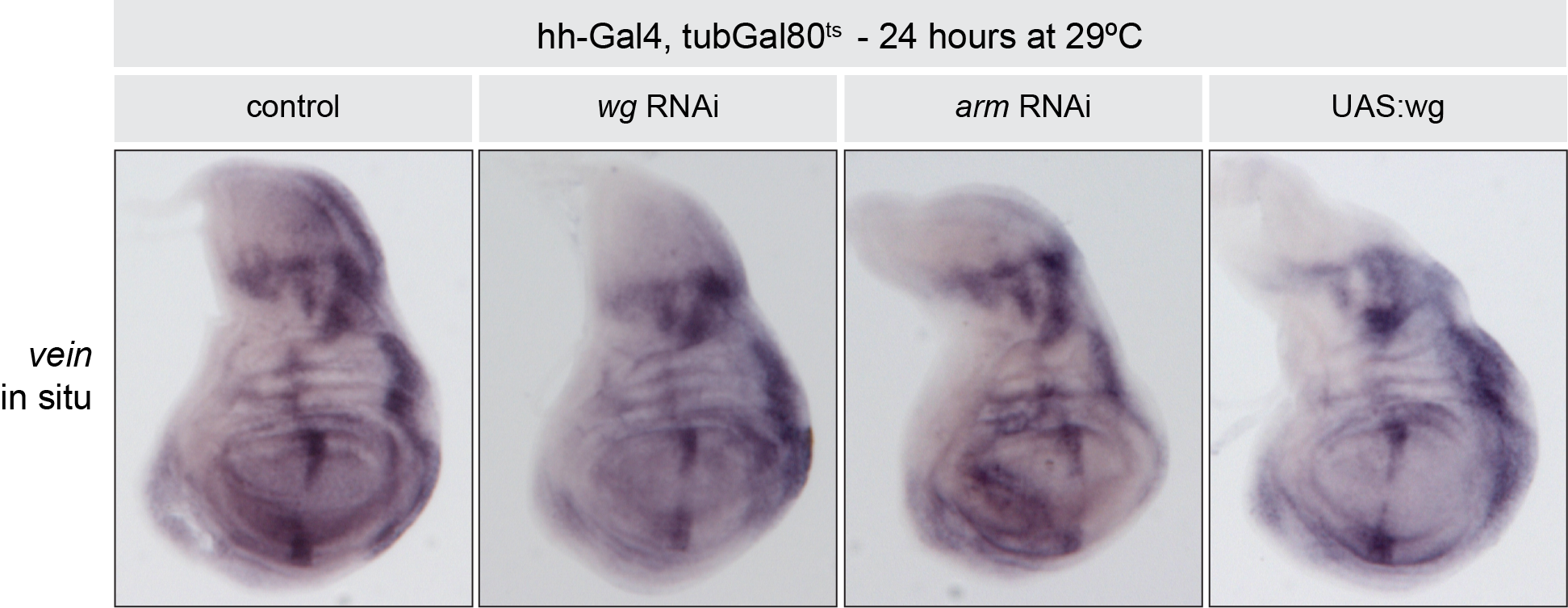
**

**Figure S9. (Related to Figure 5.) *vein* levels are not strongly modulated by varying *wg* signaling levels.** *in situ* hybridization against *vn* in the indicated genotypes.
