# Supplementary material for: Wnt signaling modulates the response to DNA damage in the Drosophila wing imaginal disc by regulating the EGFR pathway
Source: PLoS Biol. 2024 Jul 24;22(7):e3002547. doi: 10.1371/journal.pbio.3002547 (PMC11341097; doi:10.1371/journal.pbio.3002547)
Supplement: S10 Fig — UAS:p53 overexpression in the wing disc posterior causes massive apoptosis and tissue death within 24 hours (top row). This effect is not ameliorated by the coexpression of UAS-wg (bottom row). (DOCX) [file pbio.3002547.s013.docx]

**
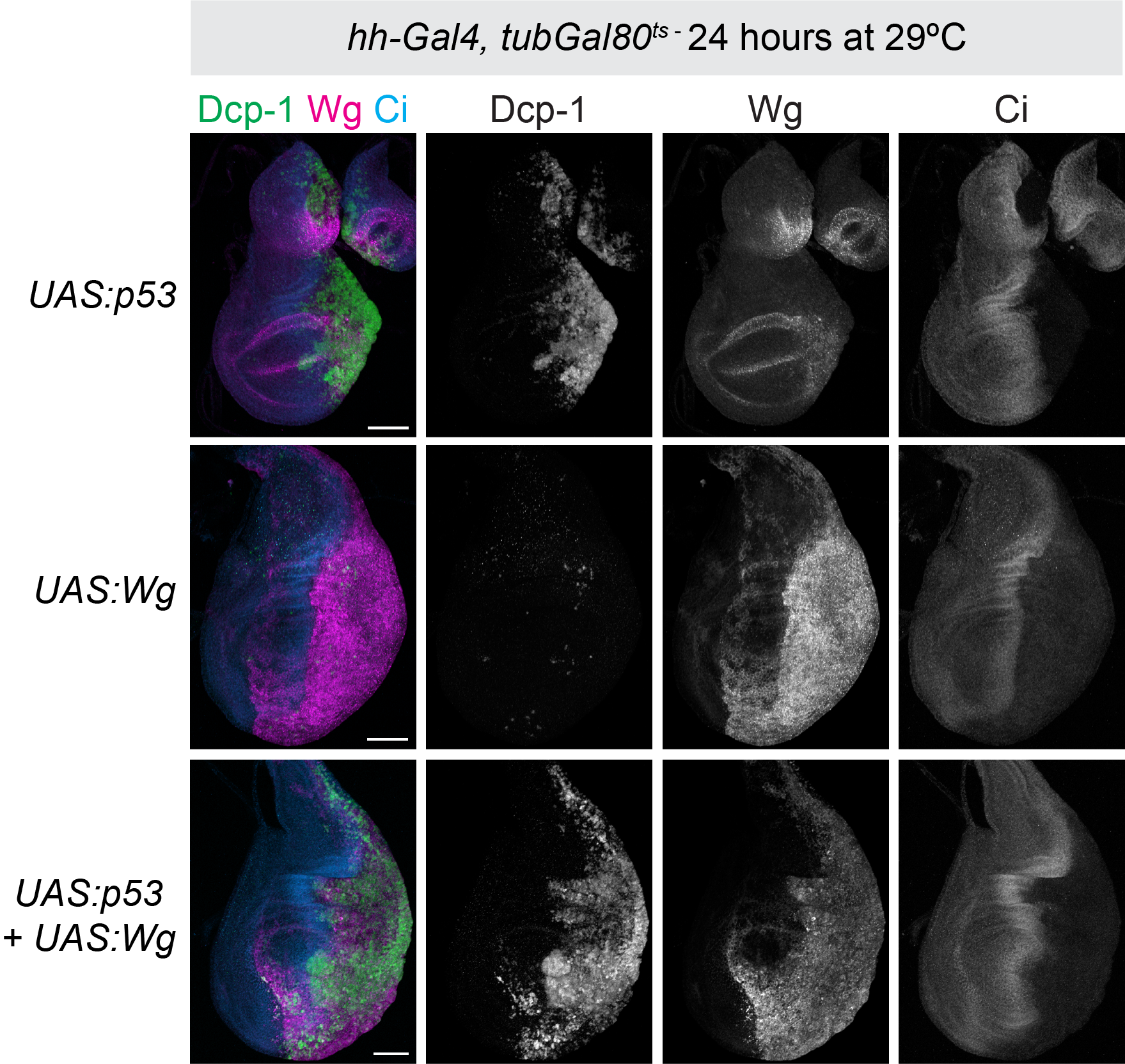
**

**Figure S10. Wg over-expression does not suppress the apoptotic effect of high levels of p53 over-expression**. *UAS:p53* over-expression in the wing disc posterior causes massive apoptosis and tissue death within 24 hours (top row.) This effect is not ameliorated by the co-expression of *UAS-wg* (bottom row.) Scale bars are 50µm, posterior is the right, and dorsal is up.
