# Supplementary material for: Wnt signaling modulates the response to DNA damage in the Drosophila wing imaginal disc by regulating the EGFR pathway
Source: PLoS Biol. 2024 Jul 24;22(7):e3002547. doi: 10.1371/journal.pbio.3002547 (PMC11341097; doi:10.1371/journal.pbio.3002547)
Supplement: Attachment — Submitted filename: 20240430-EwenCampen-response-to-reviewers.docx [file pbio.3002547.s015.docx]

May 6, 2024

Dear Dr. Alvarez-Garcia and the PLoS Biology Editorial Board:

We are very grateful to the two reviewers for their valuable and constructive feedback, and to the editorial board for the opportunity to resubmit a revised manuscript. We have now performed both additional experiments recommended by Reviewer #2, as well as an additional re-analysis requested by Reviewer #1, and below we respond to each of the comments from both reviewers. Altogether, these reviewers’ comments have greatly improved the manuscript, and all of our new experiments support the conclusions of our manuscript. Below, we respond to each of the comments in order.

**Rev. 1:
Ewen-Campen and Norbert Perrimon report that Wingless (Wnt1) signaling in Drosophila larval wing discs represses apoptosis in cells with DNA damage by inhibiting the expression of pro-apoptotic Hid. Although Wg was shown before to inhibit apoptosis by repressing the expression of pro-apoptotic Reaper in cells with DNA damage, this is the first report to link it to Hid. Moreover, the authors find that regulation occurs via transcriptional activation of rhomboid and EGFR signaling. This mode of regulation operates at intermediate levels of DNA damage induced by Cas9 and in response to DNA damaging chemicals (the data for 1000R of X-rays is less convincing for reasons given below). The identification of a new regulatory crosstalk between conserved Wg and EGFR signaling pathways in controlling apoptosis in response to damaged DNA is significant and should interest the readers of PLoS Biology. The data support most of the conclusions but there are some concerns that need to be addressed. I believe this could be done without additional experiments.**

We thank Reviewer #1 for these supportive comments and respond in detail to each concern below.

**Many of the graphs show the ratio of Dcp1 signal in P and A compartments. Because cell death is targeted to the P compartment in these experiments, the signal in the A compartment is close to zero. Dividing by a number that is close to zero can induce huge errors. Unexpected changes in the control A compartment can also add error. For example, in Fig. 2B, drug-induced Dcp1 signal in the A compartments appears lower in Arm RNAi discs than in mCherry RNAi discs, which could artificially bump up the P/A ratio. In another example, in Fig. S4, X-ray-induced Dcp1 signal in the A compartment is lower in Arm RNAi disc than in mCherry RNAi control. Differences in compartment size induced by genetic manipulations could also add error because Dcp1+ voxels will be less where there are fewer cells to begin with even if the same % of cells die. For these reasons, the authors should back up their key conclusions with a second method to normalize the signal, for example, by normalizing Dcp1+ area/volume in the P compartment to total P compartment area/volume.**

We thank Reviewer #1 for this important feedback, and below, we present a comparative re-analysis to demonstrate that **our results are robust across three different quantification methods**, including the specific recommendation of Reviewer #1, and we have also revised our manuscript as follows:

(1) We have revised the description of our method in our manuscript, to more fully explain that our method measures the *percentage* of Dcp1+ positive voxels (the 3D equivalent of a pixel) in the compartment and thus explicitly accounts for the total volume of the compartment, as recommended by Reviewer #1;
(2) Further, we have revised the description of our method to explain how we addressed the issue of “dividing by a small number” when comparing the posterior to the anterior by thresholding our images prior to quantifying Dcp1 signal such that there is a low level of “noise” or “speckling” across the tissue, which moves the denominator away from zero and thus makes small fluctuations far less impactful.
(3) We also now include in our methods section a description of the three different quantification methods that we tested (normalizing posterior:anterior, as we present in the manuscript; using the raw value of posterior % of Dcp1+ voxels; and adding the value of 1.0 to every measurement in order to push values away from zero and reduce the impact of dividing by small numbers), to emphasize that our results are robust across all three methods.

To test whether our Dcp1-based results are robust across different methods for quantifying signal, we re-analyzed the data in our Figure 1 using three different methods: (1) calculating the ratio of %Dcp1+ voxels in the posterior vs. anterior of each disc; (2) presenting the raw value of Dcp1+ positive voxels in the posterior, as suggested by Reviewer #1; and (3) adding the value of 1.0 to every value in order to reduce the influence of dividing by very small numbers. As shown below in this Figure intended for reviewers (and not included in the manuscript itself), each of the significant differences between categories are robust across these three methods – note that every important significant difference (or lack thereof) is conserved across methods, though of course the values themselves vary based on the method used.

We believe this is a useful demonstration of the robustness of these results to various ways to quantifying the effect, and we also believe that the way we quantified our results in the manuscript is the preferable way to do so, for several reasons. There is value in normalizing the posterior to the anterior to account for random variation between tissues and experiments in the amount of background signal. Further, it is more intuitive for the reader to think of the Dcp1+ signal as a ratio rather than a raw value – e.g. “five times as much cell death in the posterior as in the anterior” makes more intuitive sense than “3.5% of the cells in the posterior died in one condition.” Lastly, regarding the “add 1.0 to every value” method, while there may be some theoretical benefit to doing so to reduce variation, this forces the values on the y-axis to arbitrary units with very small incremental differences. Altogether, our results are robust to variation in quantification methods, and we believe that the changes to our Methods section that we describe above have substantially improved the manuscript.

**Related to the above, it is hard to see how images shown in some figures could produce the quantified results in the graphs. For example, in Fig. 5S, Dcp1 signal in the A and P compartments do not seem different in the discs shown (which I assume are representative), yet quantification shows nearly 2-fold differences.**

We believe this reflects three issues, which we address below.

First, whereas the data in most of our primary figures shows a large *increase* in apoptosis relative to a baseline of nearly zero apoptosis (which is very visually obvious to a reader), the irradiation and drug data inherently involves large amounts of apoptosis across the entire tissue, with either increased or decreased values in the posterior or anterior compartment – this is inherently harder to depict visually, but is accurately quantified in our data. As one example, in Figure S5B, the control panels (top row of 5SB, there is nearly uniform apoptosis across the tissue, whereas in the bottom row it is visually apparent that there is reduced apoptosis in the posterior compartment where *wg* is ectopically over-expressed.

Second, in our figures, discs are presented as 2D maximum intensity projections of 3D data, whereas the data analysis occurs across a 3D tissue. We agree that the 2D projections can make it less apparent when there is variation between compartments in some instances, but our analysis supports the conclusions.

And third, the effects of irradiation and DNA-damage drugs on *wg* RNAi or *wg-*CRISPRa tissues are indeed more modest than the effects of we observe from CRISPR-induced DSBs. For example, in Figure S4 (effect of X-rays on *wg* RNAi tissues), while the effect of *wg* RNAi on X-ray sensitivity is indeed significant (*p* = 0.0008), it is clearly relatively modest in absolute terms. To address this concern in our manuscript, we have added language to page 6 and 7 of our Results.

**Hid is clearly involved but the negative data with rpr, skl and grim RNAi could be because respective RNAi constructs are not knocking down their targets sufficiently. Can this possibility be ruled out, especially for rpr which was shown previously to be transcriptionally regulated by Wg (Ref.8)?**

We thank the reviewer for raising this issue, and we have re-worded our manuscript to address this limitation and to explicitly mention the important finding from Verghese *et al.* that Wg regulates *rpr* in the disc. We also note, however, that if Rpr was playing a significant standalone role in driving apoptosis in this context, we would expect to see apoptosis following *hid* RNAi (given that *rpr* is presumably still active in these flies), which we do not observe.

**Suggestions to improve the presentation: The sentence that 'The cyclin protein cycA is known to be required for cell cycle arrest upon DNA damage [43], and as expected cycA-RNAi strongly suppressed apoptosis in our suppressor screen (Figure S6C.)' does not make sense because Chk1 and ATR are also required for cell cycle arrest upon DNA damage and their RNAi had no effect. A more likely explanation is that cyclin A RNAi arrests cells at a stage in the cell cycle (because cyclin A is needed for normal cell cycle progression unlike Chk1 and ATR) where they are less likely to undergo apoptosis or where Cas9 works less efficiently.**

We thank Reviewer #1 (and Dr. Milan, below) for pointing out important shortcomings with our discussions of our *cycA RNAi* data. We have significantly revised our discussion of these data to explain their relevance more clearly, as *cycA* loss-of-function does not cause cell cycle arrest in the disc but in fact it causes induced endocycling (cycling between G&S without M phase) and a marked resistance to apoptosis, in the both the wing disc and multiple other tissues (e.g. PMID: **38559130, 24284207**). These data are not central to our conclusions, but we believe they warrant inclusion in our manuscript because they support the conclusion that the apoptosis we observe can be blocked by manipulations that block canonical DDR responses. However, if both reviewers and the Editorial Board believe these data are distracting and/or poorly presented, these data can be removed without affecting the conclusions of the paper.  **Ref. 8-9 are cited for the role of Wg in inhibiting radiation-induced apoptosis in the hinge region of the wing disc but 'the downstream mechanisms connecting Wnt to the DDR remain unclear, as does the question of whether Wnt signaling promotes resistance to DNA damage in other cellular contexts'. This is inaccurate because Ref. 8-9 identified transcriptional repression of pro-apoptotic gene Rpr as the mechanism by which Wg inhibits apoptosis. The authors should acknowledge this previous finding. Furthermore, a newer paper from this lab reports that Wg signaling inhibits caspase activation in regions of the wing disc outside the radioresistant hinge (PMID: 38182576). Therefore, the authors may want to update their statement about other cellular contexts.**

We thank the reviewer for identifying these instances and have corrected them in the manuscript.  **In Fig. S3E, do all panels also have Wg RNAi or Wg sgRNA? Otherwise, there are several like intergenic, yellow, and ebony that are inducing apoptosis on its own, which is contrary to the rest of the paper.**

No, the panels shown in S3E do *not* have reduced *wg* function (except the panel labeled *wg + wnt6* sgRNA), and are included to show that even supposedly non-toxic variants of UAS:Cas9 cause appreciable levels of DNA damage *in vivo.* This is important for researchers in the field to understand (that any use of catalytically active Cas9, even lower-toxicity variants, will cause apoptosis *in vivo* in a proliferative tissue like the wing disc), and also to demonstrate that there are baseline levels of apoptosis in this system that can then be used to study the effect of other pathways on the DDR.

**Please double check the references to figure numbers. For example:
Figure 3 legend refers to Figure S3, but it should be S6. In the sentence 'Only hid-RNAi suppressed apoptosis in this context, which we confirmed with two independent RNAi constructs (Figure 5A, B and Figure S8.)', 5A, B should be 4A, B.**

We thank the Reviewer for identifying these errors, and have corrected figure references throughout the manuscript. **Rev. 2: Marco Milán - note that this reviewer has signed his review
The manuscript of Ewen-Campen and Perrimon unravels a role of the Wingless signalling pathway in protecting epithelial cells to DNA damage-induced apoptosis through induction of the EGFR signalling pathway. The ms is well written, topic is timely, figures self-explanatory and the conclusions are well based on very-well designed experiments. Everything started from the initial observation that specific depletion of the Wingless ligand in the Drosophila wing epithelium enhanced CRISPR/Cas9-driven DNA damage induced cell death when targeting other Wnt ligands. From there, authors demonstrate that Wingless signalling (through the canonical pathway) dampens the apoptotic effects of DNA damage produced by several means (CRISPR/Cas9-cleavage of intergenic regions, X-rays, chemical drugs). Interestingly, Wingless appears to be also sufficient to dampen cell death when overexpressed. Through a candidate approach, authors identify elements of the DDR and EGFR pathways and present evidence that Wingless signalling reduces cell death through transcriptional induction of rhomboid (known to cleave EGFR secreted ligands) and ERK activation which will lead to the blockage of hid expression/activity. Overall, I support publication of this ms once authors have dealt with the following issues:**

We thank Dr. Milán for these supportive remarks, and for the constructive feedback that we address below.  **(1) Does Wingless signalling have any effect on DNA-damage induced cell cycle arrest?**

We agree that this is an important question, and in this manuscript we did not pursue it in detail because pilot experiments using FlyFUCCI in the context of hh-Gal4 > UAS:Cas9 + sgRNAwg+wnt6 did not reveal an coherent shift in cell cycle dynamics. However, given the known and quite complex relationship between Wnt signaling and cell cycle in the wing disc (e.g. Johnston & Edgar PMID **9665132, which examines the regulation of the G2 and G1 arrests in the zone of non-proliferation at the DV boundary**) and in many other contexts we believe it will be valuable avenue for future research and have added a reference to possible cell cycle effects in the Discussion. **(2) The results of CycA on DNA damage induced cell death bother me as I see no connection with the rescue of cell death.**

We thank Dr. Milán for these comments, which were also raised by Reviewer #1, and paste the same response here: We have significantly revised our discussion of these data to more clearly explain their relevance, as *cycA* loss-of-function does not cause cell cycle arrest in the disc but in fact it causes induced endocycling (cycling between G&S without M phase) and a marked resistance to apoptosis, in the both the wing disc and multiple other tissues (e.g. PMID: **38559130, 24284207**). These data are not absolutely central to our conclusions, but we believe warrant inclusion in our manuscript because they support the conclusion that the apoptosis we observe is occurring through the canonical DDR pathway. However, if both reviewers and the Editorial Board prefer, these data can be removed without affecting the conclusions of the paper.

**(3) Authors propose that Wg signalling regulates ERK through Rhomboid. In order to reinforce the proposed linear relationship between these elements, if would be necessary to show whether the increase in ERK levels caused by Wingless overexpression are rescued by rhomboid depletion.**

To address this important feedback, we have performed a new experiment (shown in Figure 5B and 5C.) As predicted by our model, our new data shows that *rhomboid* RNAi reduces the ERK over-expression caused by *UAS-wg.* This result strengthens our manuscript by showing that the regulation of ERK by Wg requires *rhomboid*, and we thank the reviewer for suggesting this experiment.

**(4) Labels are not correct in many figures. Ci stainings are lacking in many cases, the color of Dcp1 and Wg channels are interchanged, etc. Why the use of Ci antibody should be stated.**

We have corrected each of these issues and normalized the colors used within each figure.

**(5) I would show hid-EGFP (Figure 4C) in a single panel in B/W**

Done. In Figure 4, we have now added a row of panels showing hid-EGFP on its own in BW, in addition to the merged image.

**(6) Bergmann and Steller demonstrated (a few years ago) that ERK blocks Hid protein directly through phosphorylation. Perhaps, this should be included in the model and discussed in the paper.**

We appreciate this comment, and we have modified our model and manuscript to include this observation. **(7) Murcia et al 2019 presented evidence that DNA-damage induced ERK signaling dampens the apoptotic effects by repressing Hid. Perhaps, these data should be discussed in the paper.**

We agree, and have added this important precedent to our manuscript, which adds additional supportive evidence to our findings.

**(8) The no-effects of Bsk-DN on apoptosis worry me a lot. Have authors tested other UAS-transgenes able to block JNK more efficiently (eg. UAS-puc). Apparently, JNK is indeed involved in DNA damage induced cell death, if I recall correctly from the published literature.**

We were also surprised that reducing JNK signaling did not suppress apoptosis in this context, and we have now performed the suggested experiment: testing whether over-expression of *UAS:puc*, a negative regulator of JNK signaling, can reduce the high levels of apoptosis caused by targeting *wg* and an intergenic locus using Cas9. Using the same experimental design we used in our epistasis screen, we did *not* see a significant reduction in apoptosis when *puc* was over-expressed. These new results are presented and quantified in Supplemental Figure 7 and discussed on page 8. This result is consistent with data we presented in our original draft using a different reagent to negatively regulate JNK signaling, *Bsk[DN]*.

While we did not experimentally pursue this finding in this manuscript, we speculate that it may be a result of the fact that the DNA damage we are inducing here using Cas9, unlike X-rays, is constitutive over a 24-hour period (Cas9 is thought to persistently cut the target site unless or until it is permanently mutated to be resistant to cutting), whereas the role of JNK on the DDR in the wing disc has, to our knowledge, primarily been demonstrated in the context of acute irradiation. We interpret these results to suggest that the p53/E2F-mediated apoptosis we observe is either downstream or in parallel to JNK activity, and that the *puc-lacZ* expression we observe is likely part of a positive feedback loop initiated by high levels of apoptosis.

**(9) Please, include the complete genotypes in Table S1.**

Done. We now provide three genotype tables: Table S1 incldues full genotypes for each figure panel in the future, Table S2 includes details on sgRNA lines, and Table S3 includes the original source of each line used.
